# Herbal Granules of Heat-Clearing and Detoxifying for Children with Mild Hand, Foot, and Mouth Disease: A Bayesian Network Meta-Analysis

**DOI:** 10.1155/2022/6818406

**Published:** 2022-05-29

**Authors:** Yongcheng Sheng, Xueting Liu, Qin Wang, Yuhui Zhang, Litao Huang, Dan Hu, Pengwei Ren, Qi Hong, Deying Kang

**Affiliations:** ^1^Department of Evidence-Based Medicine and Clinical Epidemiology, West China Hospital, Sichuan University, Chengdu, Sichuan, China; ^2^Double First-class” Construction Office, West China Hospital, Sichuan University, Chengdu, Sichuan, China; ^3^Center of Biostatistics, Design, Measurement and Evaluation (CBDME), Department of Clinical Research Management, West China Hospital, Sichuan University, Chengdu, Sichuan, China; ^4^Department of Clinical Research Management, West China Hospital, Sichuan University, Chengdu, Sichuan, China

## Abstract

**Background:**

Regarding ethical considerations of randomized controlled trials (RCTs) in children, limited evidence for mild hand, foot, and mouth disease (HFMD) is available. Recently, with the increasing but result-conflicting RCTs published around herbal granules of heat-clearing and detoxifying (HGs-HD), a head-to-head comparison is urgently needed to choose a suitable therapy for clinical practice.

**Materials and Methods:**

This study was conducted according to the preferred reporting items for systematic review and meta-analysis (PRISMA) extension statement for network meta-analysis (NMA). Eight databases (Medline, Embase, and so on) and two trial registry platforms (https://www.clinicaltrials.gov and https://www.chictr.org.cn) were searched from inception to May 26, 2021. The NMA was performed using a random-effect model. The treatment hierarchy was summarized and reported as the surface under the cumulative ranking curve (SUCRA) probability values. The rankings of each HGs-HD at primary outcomes were estimated by the inverse probability weighting (IPW) approach and averaged, which presents the comprehensive improvement effect.

**Results:**

Forty-five RCTs involving 18 interventions were included that studied 5,652 children with mild HFMD. The best performance probability for improving symptoms were respectively presented in terms of fever (Xiao'er Resuqing granules, XRGs, 94.9%), rash (Xiao'er Jinqiao granules, 83.9%), hospitalization (Xiao'er Chiqiao Qingre granules, XCQGs, 92.7%), vesicles (Jinlianhua granules, 91.0%), appetite (Xiao'er Chiqiao Qingre granules, XCQGs, 86.7%), and ulcers (Kouyanqing granules, KouGs, 88.8%). Furthermore, the top 5 rankings for comprehensive improvement effect were Yanning granules (YNGs, 2.256), XCQGs (2.858), XRGs (3.270), KouGs (7.223), and Houerhuan Xiaoyan granules (HXGs, 7.597).

**Conclusions:**

This is the first NMA of HGs-HD head-to-head comparisons for children with mild HFMD. Of those, YNGs, XCQGs, XRGs, KouGs, and HXGs could be recommended as potential choices for clinical practice. Of course, the results should be interpreted with caution due to the limited high-quality RCTs.

## 1. Introduction

Large-scale outbreaks of hand, foot, and mouth disease (HFMD) have been continuously observed in the Asia-Pacific region [1]. From 2008 through 2019, a total of 763,863 HFMD cases were reported by the Chinese Center for Disease Control and Prevention, and most of these cases (753,935, 98.7%) were mild, while severe ones accounted for only 1.3% (9,928 cases, including 144 deaths) [2]. Regarding mild HFMD management, prompt recognition and treatment could prevent mild HFMD from developing into a severe one [1] and meanwhile reduce hospital admission and length of hospital stay [3].

As a broad-spectrum antiviral drug, ribavirin may have some effects on relieving symptoms of mild HFMD at an early stage but always bring severe adverse reactions, including anaphylactic shock, hematologic toxicity, and reproductive toxicity [4]. Moreover, its rational dosage for children is unclear due to the lack of evidence. Serious concerns on safety may limit its application for treating HFMD. Thus, choosing other supportive and symptomatic treatments are needed [5, 6]. In China, Chinese herbs or herbal preparations with the effect of heat-clearing and detoxicating are strongly recommended for treating HFMD [4]. A large number of randomized clinical trials (RCTs) for herbal granules of heat-clearing and detoxifying (HGs-HD) treating mild HFMD have been published [7]. However, those results are always inconsistent and even conflicting, and there is a lack of HGs-HD head-to-head comparisons [8–10].

Therefore, we conducted a systematic review with network meta-analysis (NMA) to clarify the efficacy and safety of available HGs-HD for mild HFMD in China.

## 2. Materials and Methods

This study was reported in strict compliance with PICOST framework and in accordance with the standard format, the preferred reporting items for systematic review and meta-analysis extension statement for network meta-analysis (PRISMA-NMA, see Appendix S11) [11].

### 2.1. Criteria for considering Studies for This Study

  (P) Participants: children with mild HFMD (not severe); others with complications or focused on nursing issues were excluded.
  (I) Interventions and(C) Comparators: the studies were involved that compared any of the following therapies versus any other: (1) HGs-HD + conventional treatment (HGs-HD); (2) conventional treatment (No medication, NM); (3) placebo + conventional treatment (Placebo). “HGs-HD” are defined as herbal granular preparations with heat-clearing and/or detoxifying effects, which can be conveniently taken according to certain specifications. “Conventional treatment” was defined as a therapy at least with ribavirin (regardless of dose, frequency, and form of administration).
  (O) Outcomes: (1) primary outcomes: fever clearance time; disappearance/scabbing time of rash; hospitalization/healing/treatment time; disappearance/s cabbing time of vesicles; improvement time in appetite; disappearance/healing time of ulcers. (2) Secondary outcomes: total effectiveness rate (TER); adverse effect rate (ADR).
  (S) Study design: RCT.
  (T) Time periods: treatment duration of HGs-HD is no more than 7 days.

### 2.2. Search Methods for Identification of Studies

Medline (PubMed), Embase (Ovid), Science direct, Web of science, China biology medicine disc (CBM), China national knowledge infrastructure (CNKI), Wanfang database (Wanfang data), and China science and technology journal database (VIP) were searched from inception to May 26, 2021 (see Appendix S1 for full details of the search strategy). The reference lists of the included studies, unpublished clinical trials in both the clinical trial registry (https://www.clinicaltrials.gov) and the Chinese clinical trial registry (http://www.chictr.org.cn) were also searched.

### 2.3. Data Collection

YCS and QW independently screened all studies and extracted information on population (sample size, gender, age, and course of disease), interventions (drug, dose, treatment duration, and conventional treatment) and endpoints of interest and then cross-checked the data after the extraction. Any disagreement regarding values, inconsistencies, and uncertainties were resolved by reaching consensus or by involving a third contributor (YHZ). The selection process in sufficient detail was recorded to complete a PRISMA flow diagram.

### 2.4. Assessment of Risk of Bias in Included Studies

YCS and QW independently assessed the risk of bias in 7 domains using the criteria outlined in the *Cochrane Handbook for Systematic Reviews of Interventions* [12]. Ultimately, “risk of bias” judgements across different studies for each of the listing domains were summarized.

### 2.5. Statistical Approach

#### 2.5.1. Direct Pairwise Meta-Analysis

The weighted mean difference (WMD) and its 95% confidence interval (95% CI) were selected to show the size of the effect for continuous variables. Relative risk (RR) and its 95% CI were selected to represent the magnitude of effect for binary variables. The effects of all outcomes were performed using Stata12.0 software representing as forest maps.

#### 2.5.2. Network Meta-Analysis and IPW Adjustment

NMA was conducted to estimate the effect for every class (HGs-HD) and for every individual intervention using a Bayesian Markov chain Monte Carlo method and fitted in R 4.0.2 and WinBUGS 1.4.3 [13]. The ranking probability of being at every possible rank for all interventions in every outcome was estimated. The treatment hierarchy was summarized and reported as surface under the cumulative ranking curve (SUCRA) and mean rankings. SUCRA is 1 when the treatment is certain to be the best and 0 when it is certain to be the worst [14]. To present the comprehensive improvement effect, the SUCRA rankings of primary outcomes were estimated by the inverse probability weighting (IPW) approach, and the adjusted rankings were averaged. The IPW approach estimates the differences of the rankings with the weight being the reciprocal of the SUCRA.

#### 2.5.3. Assessment of Heterogeneity and Subgroup Analysis

In the direct pairwise meta-analysis (MA), a fixed-effect model was applied if there was little statistical heterogeneity (*I*^2^ <50%), or data would be analyzed using a random-effect model (*I*^2^ ≥50%) and investigated for possible heterogeneity. Subgroup analysis was planned to be performed based on diverse HGs-HD in MA, initially assessing their effect differences in mild HFMD.

#### 2.5.4. Assessment of Inconsistency and Similarity

If the consistency test showed no inconsistency in the comparison of curative effects among intervention methods, the results were analyzed by the consistency model. Otherwise, an inconsistency model was used. The similarity assumption underlying indirect comparison MA was evaluated by comparing the distribution of clinical variables (such as age and course of disease) that could act as effect modifiers across treatment comparisons.

#### 2.5.5. Assessment of Publication Bias

For the direct pairwise MA of all outcomes, publication bias was assessed through visual inspection of a funnel plot [15].

## 3. Results

### 3.1. Description of Studies

**Figure 1:**
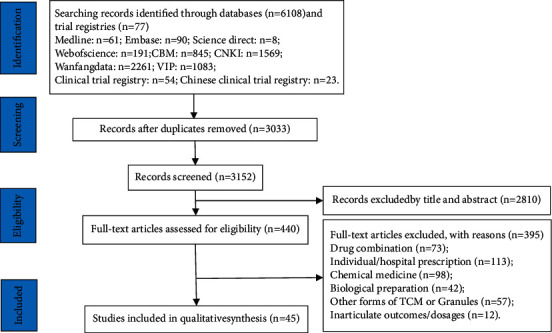
Flow diagram of studies considered for inclusion.

A total of 6,185 records from English and Chinese databases as well as trial registry platforms were identified. Eventually, 45 RCTs were involved in this study (see Appendix S2 for full reference list). These RCTs examined 18 interventions (17 HGs-HD and NM) among 5,652 children (mean age, about 3 years; range, 0–12; mean course of disease, around 2.7 days; and range 0–13). Publication years varied from 2009 to 2021 (median, 2016). The number of participants included in these RCTs ranged from 56 [16] to 380 [17] (median, 108).  Figure 1 presents the screening flow of studies (see Appendix S3 and S4 for full details of each included RCT). The information for HGs-HD in detail is shown in Appendix S5.

### 3.2. Risk of Bias in Included Studies

**Figure 2:**
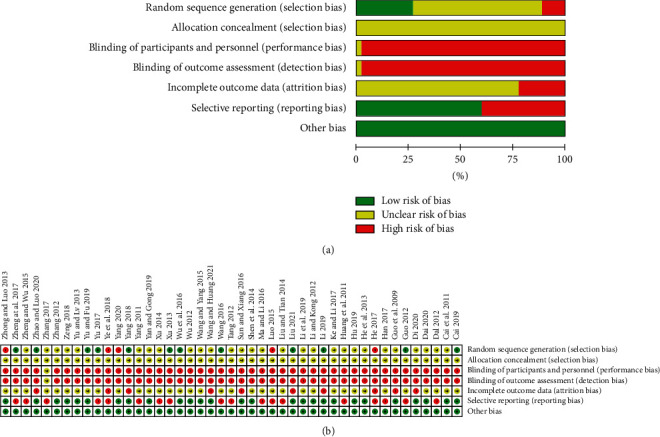
Risk of bias for included studies. (a) Risk of bias graph; (b) risk of bias summary.

Generally, the quality of the included RCTs was low to moderate as presented in Figure 2. ① Selection bias (random sequence generation): twelve RCTs (26.7%) used random number table (low risk), and 28 RCTs (62.2%) just referred to “random” with no detailed method of randomization (unclear risk) while 5 RCTs (11.1%) grouped patients according to the time of admission (high risk). ② Selection bias (allocation concealment): because information on allocation concealment was not observed in all RCTs (100.0%), this domain was evaluated as “unclear risk.” ③ Performance bias and ④ Detection bias: only one RCT was deemed as “unclear risk” as for referring to double blinding, while the others (97.8%) were evaluated as “high risk” regarding blinding of participants, personnel, and outcome assessors because the control group did not use a placebo. ⑤ Attrition bias: ten RCTs (22.2%) reported the probably existing dropout (high risk) and the remaining (77.8%) were unclear about it. ⑥ Reporting bias: because all RCTs provided no information about trial registry, we assessed RCTs which reported all outcomes described in the “Methods” part as low risk, otherwise high risk would be rated. Finally, 27 RCTs (60.0%) were high risk and 18 RCTs (40.0%) were low risk in this domain. ⑦ Other bias: all RCTs (100.0%) were assessed low risk as they reported baseline comparability.

### 3.3. Results of Direct Pairwise Meta-analyses

#### 3.3.1. Results of Primary Outcomes

The results of direct pairwise MA in a random-effect model for primary outcomes are, respectively, shown in Table 1 and Appendix S6.

Overall, HGs-HD were associated with a significant decrease in the pooled analysis of 44 RCTs with 5,566 patients in fever clearance time (WMD = −1.32 days; 95% CI: −1.63, −1.01), of 30 RCTs with 3,834 patients in disappearance/scabbing time of rash (WMD = −1.82 days; 95% CI: −2.49, −1.14), of 16 RCTs with 1,931 patients in hospitalization/healing/treatment time (WMD = −1.72 days; 95% CI: −1.98, −1.47), of 13 RCTs with 1,552 patients in disappearance/scabbing time of vesicles (WMD = −1.71 days; 95% CI: −2.08, −1.34), of 10 RCTs with 1,125 patients in improvement time in appetite (WMD = −1.26 days; 95% CI: −1.65, −0.87), and of 15 RCTs with 2,136 patients in disappearance/healing time of ulcers (WMD = −1.98 days; 95% CI: −2.41, −1.56) compared with NM. In the subgroup analysis, only Ertong Huichun granules (EHGs) and Xiao'er Chaigui Tuire granules (XCTGs) in fever, Kanggan granules (KangGs) in rash, Bairui granules in hospitalization, and Qingkailing granules in appetite showed no significant difference in comparison with NM.

#### 3.3.2. Results of Secondary Outcomes

TER and ADR for HGs-HD versus NM to treat mild HFMD derived from direct pairwise MA are shown in Table 1 and Appendix S6.

In terms of TER, HGs-HD showed an overall improved effect (RR: 1.20; 95% CI: 1.16, 1.24) compared with NM in the pooled analysis of 40 RCTs with 5,060 patients. In the subgroup analysis, most HGs-HD showed a significant difference except KangGs, Kouyanqing granules (KouGs), XCTGs, and XJGs. Regarding safety, there was no significant difference (RR: 0.82; 95% CI: 0.56, 1.20) for HGs-HD compared with NM in the pooled analysis of 21 RCTs with 2,199 patients. In the subgroup analysis, there was also no significant difference. In addition, adverse reactions of the included RCTs in detail are shown in Appendix S7. And 46 (4.06%) participants in the HGs-HD group and 52 (4.66%) participants in the NM group had nausea, vomiting, diarrhea, anorexia, rash, pruritus, abnormal blood routine, and so on.

### 3.4. Results of Network Meta-Analyses and Comprehensive Evaluations

**Figure 3:**
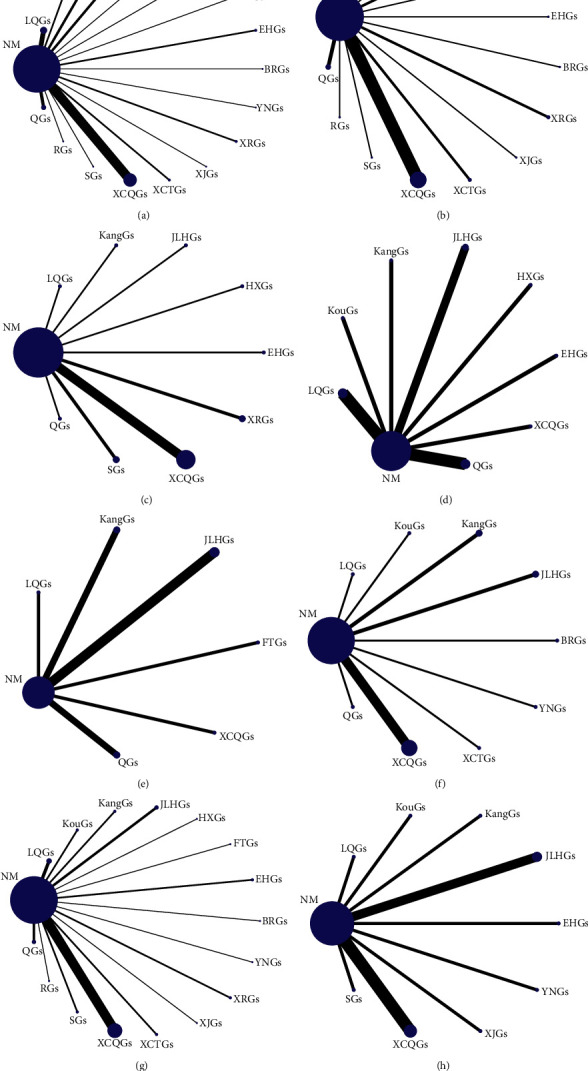
Evidence networks. (a) Fever clearance time; (b) disappearance/scabbing time of rash; (c) hospitalization/healing/treatment time; (d) disappearance/scabbing time of vesicles; (e) improvement time in appetite; (f) disappearance/healing time of ulcers; (g) total effectiveness rate; (h) adverse effect rate; NM: no medication; BRGs: Bairui granules; EHGs: Ertong Huichun granules; FTGs: Fangfeng Tongsheng granules; HXGs: Houerhuan Xiaoyan granules; JLHGs: Jinlianhua granules; JBGs: Jinye Baidu granules; KangGs: Kanggan granules; KouGs: Kouyanqing granules; LQGs: Lianhua Qingwen granules; QGs: Qingkailing granules; RGs: Reduping granules; SGs: Shanlameiye granules; XCTGs: Xiao'er Chaigui Tuire granules; XCQGs: Xiao'er Chiqiao Qingre granules; XJGs: Xiao'er Jinqiao granules; XRGs: Xiao'er Resuqing granules; and YNGs: Yanning granules.

Evidence networks for primary outcomes are shown in Figures 3(a)∼3(f) and the ranks of interventions based on SUCRA analysis are displayed in Table 2 and Appendix S8. All HGs-HD treatment options surpassed NM.

The top five HGs-HD for fever clearance time were Xiao'er Resuqing granules (XRGs, 94.9%), Yanning granules (YNGs, 88.6%), XCQGs (86.0%), KouGs (79.7%), KangGs (63.7%). Similarly, with the disappearance/scabbing time of rash, XJGs (83.9%) were ranked the highest, followed by XCQGs (83.0%), SGs (78.8%), Houerhuan Xiaoyan granules (HXGs, 78.4%), and XRGs (77.5%). XCQGs (92.7%) showed the most favorable response for hospitalization/healing/treatment time, followed by XRGs (86.9%), EHGs (68.6%), SGs (63.4%), and Qingkailing granules (QGs, 50.9%). Furthermore, Jinlianhua granules (JLHGs, 91.0%) were the best intervention to improve the disappearance/scabbing time of vesicles, followed by KouGs (90.6%), HXGs (76.8%), XCQGs (75.9%), and KangGs (67.1%). As for improvement time in appetite, the top five were XCQGs (86.7%), JLHGs (75.0%), KangGs (69.6%), QGs (65.4%), and Lianhua Qingwen granules (LQGs, 49.3%). In terms of disappearance/healing time of ulcers, KouGs (88.8%) achieved the most positive effect, followed by YNGs (88.7%), XCQGs (80.1%), LQGs (61.1%), and Bairui granules (BRGs, 57.4%).

Through comprehensive evaluations of primary outcomes by IPW approach, the top five HGs-HD were YNGs (2.256), XCQGs (2.858), XRGs (3.270), KouGs (7.223), and HXGs (7.597).

**Figure 4:**
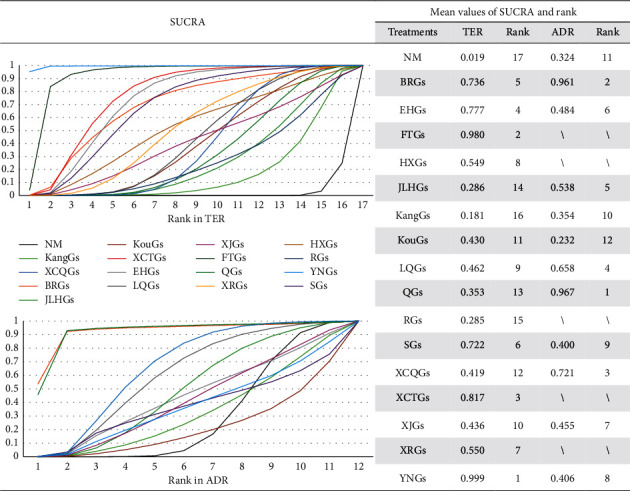
SUCRA and ranking for TER and ADR. The best results were marked as bold font; “\” denotes “not included”; TER: total effectiveness rate; ADR: adverse effect rate; NM: no medication; BRGs: Bairui granules; EHGs: Ertong Huichun granules; FTGs: Fangfeng Tongsheng granules; HXGs: Houerhuan Xiaoyan granules; JLHGs: Jinlianhua granules; JBGs: Jinye Baidu granules; KangGs: Kanggan granules; KouGs: Kouyanqing granules; LQGs: Lianhua Qingwen granules; QGs: Qingkailing granules; RGs: Reduping granules; SGs: Shanlameiye granules; XCTGs: Xiao'er Chaigui Tuire granules; XCQGs: Xiao'er Chiqiao Qingre granules; XJGs: Xiao'er Jinqiao granules; XRGs: Xiao'er Resuqing Granules; and YNGs: Yanning granules.

Evidence networks and results of NMA for secondary outcomes are shown in Figures 3(h) and 4. On the basis of SUCRA for TER, YNGs (99.9%) and Fangfeng Tongsheng granules (FTGs, 98.0%) had the greatest therapy while XCTGs (81.7%), EHGs (77.7%), BRGs (73.6%), and Shanlameiye granules (SGs, 72.2%) could be the second-best interventions. And XRGs (55.0%), HXGs (54.9%), LQGs (46.2%), XJGs (43.6%), KouGs (43.0%), XCQGs (41.9%), QGs (35.3%), JLHGs (28.6%), Reduping Granules (RGs, 28.5%), and KangGs (18.1%) would be the third-best ones while NM could be the worst (0.9%). Similarly, with ADR, QGs (96.7%), and BRGs (96.1%) were ranked the best safety, followed by XCQGs (72.1%) and LQGs (65.8%), then followed by JLHGs (53.8%), EHGs (48.4%), XJGs (45.5%), YNGs (40.6%), SGs (40.0%), KangGs (35.4%), NM (32.4%), and KouGs (23.2%).

### 3.5. Inconsistency, Similarity, and Publication Bias

The evaluation of the inconsistency between direct and indirect comparisons was unnecessary because a loop connecting the three arms did not exist in our study. Assessment of similarity by scatter diagram (see Appendix S9) indicated that the mean age and mean course of disease were almost the same between control and test groups while a little discrepant across treatment comparisons despite some unreported courses of disease in some RCTs. Upon visual inspection, the funnel plots were symmetrical in fever clearance time, improvement time in appetite, disappearance/healing time of ulcers, TER, and ADR (Appendix S10: Figures S10(a), S10(e)–S10(f)). And there were no publication biases. The funnel plots were significantly asymmetrical in disappearance/scabbing time of rash, hospitalization/healing/treatment time, and disappearance/scabbing time of vesicles (Appendix S10: Figures S10(b)–S10(d)), and they showed some publication biases.

## 4. Discussion

### 4.1. Main Findings

To our knowledge, it was the first NMA of HGs-HD for treating mild HFMD. All HGs-HD showed better efficacy surpassing NM. Of those, YNGs, XCQGs, XRGs, KouGs, and HXGs could be recommended as potential interventions for clinical practice.

### 4.2. Interpretations of Findings

As reported, the heat-clearing and detoxifying therapy has been proved to reduce the progressive rate of mild HFMD and healing time of rash or oral ulcer [18, 19], and HGs-HD were superior to placebo in resolution of fever symptom [20]. They could relieve the clinical symptoms, such as fever, rash, vesicles, and ulcers, by regulating the human immune response, boosting the body's resistance to inflammation, and preventing infective pathogens from invasion [21, 22]. In our study, HGs-HD also shortened the symptom improvement time of 1.26∼1.98 days. To better improve clinical symptoms, choices of HGs-HD also could rely on the patient's conditions considering their different effects and functions.

In the SUCRA analysis, ranking the highest in fever clearance time was XRGs, in which Bupleuri Radix, the most important traditional Chinese crude drug for treating intermittent fever [23, 24], showed that its aqueous extract took the antipyretic effect by causing a dose-dependent decrease in the content of inflammatory mediators IL-1*β*, IL-6, TNF-*α*, and PGE 2 in the blood, decreasing the content of cAMP in the hypothalamus and AVP in the brain ventral septum, and increasing the content of plasma AVP [25]. As for the disappearance/scabbing time of the rash, XJGs was the best one and its *Lonicerae japonicae* flos could improve cetuximab-induced acneiform rash by external application [26, 27]. In term of disappearance/scabbing time of vesicles, JLHGs was the best intervention, and the orientin of *Trollius chinensis* took effect by attaching to the cell surface to prevent the adsorption and entry of Coxsackievirus B3 [28]. To shorten the disappearance/healing time of ulcers, KouGs achieved the most positive effect, and studies showed it attenuated the symptoms of oral ulcers of rats worsened by sleep deprivation probably through the regulation of the neuro-immuno-endocrine system, oxidative stress levels, and tryptophan metabolism [29], in which 16 ingredients played a vital role [30, 31]. Similarly, in terms of improvement time in appetite and hospitalization/healing/treatment time, XCQGs performed the best effect. The metabolism of Sojae Semen Praeparatum of XCQGs could help recovery of appetite by regulating intestinal microflora structure. The relative abundance of some microbial genus, which are beneficial to human health are significantly upregulated, while that of some conditional pathogenic microbial genus downregulated on the contrary [32].

### 4.3. Comparisons with Existing Literature

As for some meta-analyses of single HGs-HD, XCTGs [9, 10], XCQGs [8,10] and LQGs [33] showed better efficacy and safety than the control group for treating mild HFMD, but the included interventions were apparently inconsistent, which made the results incredible. Although a recent individual patient data MA showed the heat-clearing and detoxifying therapy could reduce the progressive rate of mild HFMD [34], the five interventions of different dosage forms, including effervescent tablets, oral solutions, and herbal injections, were incomparable as well. With the wide application of Chinese herbal injections, some studies with NMAs in HFMD were reported [35, 36]. However, these injections could be unsuitable for mild HFMD considering the safety of intravenous administration, for the complex compounds could accelerate the risk of cardiovascular events due to their straight entrance into the blood circulation [7], which would result in high fever and abnormal shivering. They occupied nearly 50% of ADR in Chinese medicine [37, 38]. Instead, oral medications like HGs-HD are more suitable for children [39], since they are safer during the long clinical experiences [40].

### 4.4. Strengths, Limitations, and Future Research

This NMA formulated a strict PICOST framework [41]. All included RCTs were under the definite treatment of ribavirin with similar baseline and intervention characteristics to reduce the interference of clinical heterogeneity. More importantly, this NMA made up for the lack of HGs-HD direct comparisons in mild HFMD. And our study used objective improvement time as primary outcomes instead of composite endpoints such as TER, which made the outcome measures more sensitive and scientific [42]. In addition, the IPW approach was conducted to deal with missing data in SUCRA rankings, which help comprehensively evaluate the effects of HGs-HD. Regarding HGs-HD, novel Chinese patent medications are manufactured by low-temperature spray drying technology after the decoction of Chinese herbal preparations. Their usages are as easy and convenient as making coffee, and they are welcomed especially in children as a result of their advantages of being locally accessible, convenient for storage, easy to carry, pleasant smell, and satisfactory drug compliance.

However, certain limitations of this study have to be noted. First of all, the included trial number of most HGs-HD except LQGs (6 RCTs), QGs (4 RCTs), and XCQGs (12 RCTs) is usually very small (1∼3 RCTs), which may be likely to cause false positive or negative conclusions. Only if the sample size was increased and the number of RCTs focused on different kinds of HGs-HD were balanced, the credibility of this NMA would be enhanced [43]. Secondly, time indicators as primary outcomes were objective and sensitive while they could also come along with high heterogeneity as a continuous variable, which was similar to previous studies [8, 9]. Limited by the included trial number of each HGs-HD, other methods to further decrease heterogeneity could not be conducted in our study. Thirdly, since traditional Chinese medicine (TCM) is a complex mixture of multiple components [44], the effects of HGs-HD were not fully enough to attribute to a single Chinese herb or ingredient, and the mechanism and material basis were needed to be further explored [40]. Fourthly, given the nature of the indirect comparisons of NMA, it remains a surrogate for head-to-head RCTs. Fifthly, the 45 RCTs were all conducted in China with a little risk of publication bias. Our study had not been registered, but we still strictly followed the professional reporting specification to reduce bias as far as possible. Sixthly, the total quality of the included RCTs was not high under a strict evaluation, which was similar to previous NMAs [45, 46], so we urgently make a recommendation that RCTs about children should be registered ahead of time to improve the transparency of the process and the methodological quality of studies.

## 5. Conclusions

In summary, it was the first NMA that investigated HGs-HD head-to-head comparisons for treating mild HFMD. HGs-HD all showed an improved effect in clinical symptoms compared with NM. Of those, YNGs, XCQGs, XRGs, KouGs, and HXGs could be recommended as potential interventions. The clinical choice of HGs-HD should also be made considering their different effects and functions. Meanwhile, the results should be interpreted with caution, and more high-quality RCTs are needed to fully illustrate the efficacy and safety of HGs-HD.

## Figures and Tables

**Table 1:** Results of direct pairwise meta-analysis for all outcomes.

Comparisons	WMD (95% CI)						RR (95% CI)	
	A	B	C	D	E	F	G	H
BRGs vs NM	−1.06 (−1.24, −0.88)	−1.48 (−1.74, −1.22)	−1.71 (−2.62, −0.80)	−0.36 (−0.81, 0.08)	—	−1.79 (−2.11, −1.47)	1.36 (1.10, 1.70)	—
EHGs vs NM	−0.81 (−1.82, 0.20)	−1.25 (−2.03, −0.47)	—	—	—	—	1.36 (1.14, 1.63)	1.00 (0.15, 6.82)
FTGs vs NM	−0.60 (−0.86, −0.34)	—	—	—	−0.67 (−0.93, −0.41)	—	1.82 (1.36, 2.43)	—
HXGs vs NM	−1.39 (−1.88, −0.90)	−2.56 (−3.13, −1.99)	−1.23 (−1.75, −0.71)	−2.17 (−2.65, −1.69)	—	—	1.24 (1.02, 1.52)	—
JBGs vs NM	−1.29 (−1.60, −0.98)	—	—	—	—	—	—	—
JLHGs vs NM	−1.12 (−1.23, −1.01)	—	−1.10 (−1.25, −0.95)	−2.54 (−3.40, −1.68)	−1.37 (-2.01, -0.73)	−1.45 (−1.71, −1.20)	1.12 (1.03, 1.23)	0.73 (0.30, 1.77)
KangGs vs NM	−1.36 (−1.78, −0.94)	−0.66 (−1.39, 0.06)	−0.96 (−1.31, −0.61)	−1.95 (−2.23, −1.67)	−1.31 (−2.23, −0.39)	−1.00 (−2.56, 0.56)	1.10 (0.96, 1.26)	1.11 (0.49, 2.54)
KouGs vs NM	−1.69 (−2.37, −1.02)	−1.25 (−1.54, −0.96)	—	−2.53 (−2.73, −2.33)	—	−3.13 (−3.37, −2.89)	1.18 (0.96, 1.45)	2.50 (0.50, 12.51)
LQGs vs NM	−0.76 (−1.01, −0.52)	−1.12 (−1.27, −0.97)	−1.20 (−2.01, −0.39)	−1.24 (−1.47, −1.00)	−0.83 (−0.96, −0.70)	−1.91 (−2.05, −1.77)	1.19 (1.11, 1.28)	0.51 (0.19, 1.38)
QGs vs NM	−1.00 (−1.37, −0.63)	−1.12 (−1.63, −0.61)	−1.25 (−1.58, −0.92)	−1.43 (−2.08, −0.78)	−1.19 (−2.77, 0.39)	−1.48 (−2.08, −0.88)	1.16 (1.01, 1.32)	—
RGs vs NM	−0.85 (−1.22, −0.48)	−1.80 (−2.66, −0.94)	—	—	—	—	1.12 (1.00, 1.25)	—
SGs vs NM	−1.24 (−1.76, −0.72)	−2.57 (−3.13, −2.01)	−1.53 (−1.84, −1.22)	—	—	—	1.34 (1.09, 1.63)	3.00 (0.13, 71.92)
XCTGs vs NM	−0.86 (−2.42, 0.69)	−1.23 (−2.70, −0.24)	—	—	—	−1.10 (−1.37, −0.83)	1.54 (0.87, 2.73)	—
XCQGs vs NM	−1.77 (−2.21, −1.32)	−2.45 (−3.45, −1.45)	−2.23 (−2.78, −1.69)	−2.14 (−2.36, −1.92)	−2.03 (−2.43, −1.64)	−2.45 (−3.12, −1.78)	1.17 (1.12, 1.22)	0.52 (0.21, 1.30)
XJGs vs NM	−1.10 (−1.26, −0.94)	−2.80 (−2.92, −2.68)	—	—	—	—	1.18 (0.98, 1.43)	0.81 (0.27, 2.47)
XRGs vs NM	−2.20 (−2.43, −1.97)	−2.39 (−2.80, −1.97)	−2.12 (−2.47, −1.77)	—	—	—	1.23 (1.11, 1.38)	—
YNGs vs NM	−2.05 (−2.24, −1.86)	—	—	—	—	−3.13 (−3.51, −2.75)	—	1.71 (0.16, 17.98)
HGs-HD vs NM	−1.32 (−1.63, −1.01)	−1.82 (−2.49, −1.14)	−1.72 (−1.98, −1.47)	−1.71 (−2.08, −1.34)	−1.26 (−1.65, −0.87)	−1.98 (−2.41, −1.56)	1.20 (1.16, 1.24)	0.82 (0.56, 1.20)

WMD, weighted mean difference; 95% CI, 95% confidence interval; RR, relative risk; A, fever clearance time; B, disappearance/scabbing time of rash; C, hospitalization/healing/treatment time; D, disappearance/scabbing time of vesicles; E, improvement time in appetite; F, disappearance/healing time of ulcers; G, total effectiveness rate; H, adverse effect rate; HGs-HD, herbal granules of heat-clearing and detoxifying; NM, no medication; BRGs, Bairui granules; EHGs, Ertong Huichun granules; FTGs, Fangfeng Tongsheng granules; HXGs, Houerhuan xiaoyan granules; JLHGs, Jinlianhua granules; JBGs, Jinye Baidu granules; KangGs, Kanggan granules; KouGs, Kouyanqing granules; LQGs, Lianhua Qingwen granules; QGs, Qingkailing granules; RGs, Reduping granules; SGs, Shanlameiye granules; XCTGs, Xiao'er Chaigui Tuire granules; XCQGs, Xiao'er Chiqiao Qingre granules; XJGs, Xiao'er Jinqiao granules; XRGs, Xiao'er Resuqing granules; YNGs, Yanning granules. The results with statistical significance were marked as bold font; “—” denotes “not included.”

**Table 2:** SUCRA and ranking for primary outcomes as well as comprehensive ranking.

Treatments	*A*	Rank	*B*	Rank	*C*	Rank	*D*	Rank	*E*	Rank	*F*	Rank	Comprehensive ranking
YNGs	0.886	2	—	—	—	—	—	—	—	—	0.887	2	2.256
XCQGs	0.860	3	0.830	2	0.927	1	0.759	4	0.867	1	0.801	3	2.858
XRGs	0.949	1	0.775	5	0.869	2	—	—	—	—	—	—	3.270
KouGs	0.797	4	0.438	9	—	—	0.906	2	—	—	0.888	1	7.223
HXGs	0.634	6	0.784	4	0.503	6	0.768	3	—	—	—	—	7.597
SGs	0.563	8	0.788	3	0.634	4	—	—	—	—	—	—	8.110
XJGs	0.498	9	0.839	1	—	—	—	—	—	—	—	—	9.633
JLHGs	0.492	10	—	—	0.445	8	0.910	1	0.750	2	0.478	7	11.341
JBGs	0.590	7	—	—	—	—	—	—	—	—	—	—	11.855
BRGs	0.477	11	0.505	7	—	—	—	—	—	—	0.574	5	15.207
QGs	0.454	12	0.385	12	0.509	5	0.444	6	0.654	4	0.487	6	16.556
KangGs	0.637	5	0.246	13	0.390	9	0.671	5	0.696	3	0.338	9	20.373
LQGs	0.307	16	0.393	11	0.490	7	0.359	7	0.493	5	0.611	4	21.765
RGs	0.378	13	0.593	6	—	—	—	—	—	—	—	—	22.243
XCTGs	0.348	14	0.425	10	—	—	—	—	—	—	0.377	8	28.298
EHGs	0.336	15	0.439	8	0.686	3	0.147	8	—	—	—	—	30.382
FTGs	0.267	17	—	—	—	—	—	—	0.429	6	—	—	38.815
NM	0.026	18	0.060	14	0.046	10	0.036	9	0.112	7	0.058	10	269.821

Note: The top 5 results were marked as bold font; “—” denotes “not included”; A, fever clearance time; B, disappearance/scabbing time of rash; C, hospitalization/healing/treatment time; D, disappearance/scabbing time of vesicles; E, improvement time in appetite; F, disappearance/healing time of ulcers; NM, no medication; BRGs, Bairui granules; EHGs: Ertong Huichun granules; FTGs, Fangfeng Tongsheng granules; HXGs, Houerhuan Xiaoyan granules; JLHGs, Jinlianhua granules; JBGs, Jinye Baidu granules; KangGs, Kanggan granules; KouGs, Kouyanqing granules; LQGs, Lianhua Qingwen granules; QGs, Qingkailing granules; RGs, Reduping granules; SGs, Shanlameiye granules; XCTGs, Xiao'er Chaigui Tuire granules; XCQGs, Xiao'er Chiqiao Qingre granules; XJGs, Xiao'er Jinqiao granules; XRGs, Xiao'er Resuqing granules; YNGs, Yanning granules.

## Data Availability

The data used to support the findings of this study are included within the supplementary information files.

## Abbreviations

95% CI: 95% confidence interval
ADR: Adverse effect rate
BRGs: Bairui granules
EHGs: Ertong huichun granules
FTGs: Fangfeng tongsheng granules
CPGs: Clinical practice guidelines
HFMD: Hand, foot, and mouth disease
HGs-HD: Herbal granules for heat-clearing and detoxifying
HXGs: Houerhuan xiaoyan granules
IPW: Inverse probability weighting
JBGs: Jinye baidu granules
JLHGs: Jinlianhua granules
KangGs: Kanggan granules
KouGs: Kouyanqing granules
LQGs: Lianhua qingwen granules
MA: Meta-analysis
NM: No medication
NMA: Network meta-analysis
PICOST: P: patient: problem or population; I: intervention; C: comparison: control or comparator; O: outcome; S: study design; T: time periods
QGs: Qingkailing granules
RCT: Randomized clinical trial
RGs: Reduping granules
RR: Relative risk
SGs: Shanlameiye granules
SUCRA: Surface under the cumulative ranking curve
TCM: Traditional Chinese medicine
TER: Total effectiveness rate
WMD: Weighted mean difference
XCQGs: Xiao'er chiqiao qingre granules
XCTGs: Xiao'er chaigui tuire granules
XJGs: Xiao'er jinqiao granules
XRGs: Xiao'er resuqing granules
YNGs: Yanning granules.

## Supplementary Materials

Supplementary Materials Supplementary materials associated with this study can be found in Supplement (Appendix S1–S11).
