# Supplementary material for: Herbal Granules of Heat-Clearing and Detoxifying for Children with Mild Hand, Foot, and Mouth Disease: A Bayesian Network Meta-Analysis
Source: Evid Based Complement Alternat Med. 2022 May 29;2022:6818406. doi: 10.1155/2022/6818406 (PMC9168089; doi:10.1155/2022/6818406)
Supplement: Supplementary Materials — associated with this study can be found in Supplement (Appendix S1–S11). [file 6818406.f1.pdf]

Herbal Granules of Heat-clearing and Detoxifying for Children with Mild Hand, Foot and Mouth Disease: A Bayesian Network Meta-analysis

Supplementary appendix to the manuscript

Contents of additional appendix

Appendix S1 - 1 -
Search strategy and results - 1 -
Appendix S2 - 19 -
References for included studies - 19 -
Appendix S3 - 23 -
Characteristics of included studies - 23 -
Appendix S4 - 29 -
Baseline characteristics of age and course among all studies - 29 -
Appendix S5 - 31 -
Information of HGs-HD - 31 -
Appendix S6 - 37 -
Direct pairwise meta-analysis of all outcomes for mild HFMD. - 37 -
Appendix S7 - 42 -
Adverse reactions of HGs-HD in the included RCTs - 42 -
Appendix S8 - 44 -
Radar map of ranking of treatment options relative to six primary outcomes based on SUCRA - 44 -
Appendix S9 - 46 -
Assessment of similarity results on mean age and mean course of disease among 45 studies - 46 -
Appendix S10 - 48 -
Comparison-adjusted funnel plot for mild HFMD - 48 -
Appendix S11 - 51 -
PRISMA NMA Checklist - 51 -

Appendix S1

Search strategy and results

Table S1. Number of citations by each database and trial register searched

Databases and trial registers	Citations
**Databases**	
Medline	61
Embase	90
Science direct	8
Web of science	191
CBM	845
CNKI	1569
Wanfang data	2261
VIP	1083
**Total databases**	**6108**
**Trial registers**	
Clinical trial registry	54
Chinese clinical trial registry	23
**Total trial registers**	**77**

Search strategy for Ovid-Medline

1. HFMD.sh.
2. HFMD.ti.
3. HFMD.ab.
4. HFMD.kf.
5. HFMD.tc.
6. HFMD.kw.
7. (Hand Foot and Mouth Disease).sh.
8. (Hand Foot and Mouth Disease).ti.
9. (Hand Foot and Mouth Disease).ab.
10. (Hand Foot and Mouth Disease).kf.
11. (Hand Foot and Mouth Disease).tc.
12. (Hand Foot and Mouth Disease).kw.
13. or/1-12
14. (Chinese drug therapy).sh.
15. (Chinese drug therapy).ti.
16. (Chinese drug therapy).ab.
17. (Chinese drug therapy).kf.
18. (Chinese drug therapy).tc.
19. (Chinese drug therapy).kw.
20. (Chinese herb Treatment).sh.
21. (Chinese herb Treatment).ti.
22. (Chinese herb Treatment).ab.
23. (Chinese herb Treatment).kf.
24. (Chinese herb Treatment).tc.
25. (Chinese herb Treatment).kw.
26. (Traditional Chinese medicin).sh.
27. (Traditional Chinese medicin).ti.
28. (Traditional Chinese medicin).ab.
29. (Traditional Chinese medicin).kf.

30. (Traditional Chinese medicin).tc.
31. (Traditional Chinese medicin).kw.
32. (Chinese medicine).sh.
33. (Chinese medicine).ti.
34. (Chinese medicine).ab.
35. (Chinese medicine).kf.
36. (Chinese medicine).tc.
37. (Chinese medicine).kw.
38. (Chinese patent drug).sh.
39. (Chinese patent drug).ti.
40. (Chinese patent drug).ab.
41. (Chinese patent drug).kf.
42. (Chinese patent drug).tc.
43. (Chinese patent drug).kw.
44. (Chinese patent medicine).sh.
45. (Chinese patent medicine).ti.
46. (Chinese patent medicine).ab.
47. (Chinese patent medicine).kf.
48. (Chinese patent medicine).tc.
49. (Chinese patent medicine).kw.
50. (Chinese traditional medicine).sh.
51. (Chinese traditional medicine).ti.
52. (Chinese traditional medicine).ab.
53. (Chinese traditional medicine).kf.
54. (Chinese traditional medicine).tc.
55. (Chinese traditional medicine).kw.
56. (Chinese preparation).sh.
57. (Chinese preparation).ti.
58. (Chinese preparation).ab.
59. (Chinese preparation).kf.
60. (Chinese preparation).tc.
61. (Chinese preparation).kw.
62. (Preparation Chinese materia medica).sh.
63. (Preparation Chinese materia medica).ti.
64. (Preparation Chinese materia medica).ab.
65. (Preparation Chinese materia medica).kf.
66. (Preparation Chinese materia medica).tc.
67. (Preparation Chinese materia medica).kw.
68. (Tcm preparation).sh.
69. (Tcm preparation).ti.
70. (Tcm preparation).ab.
71. (Tcm preparation).kf.
72. (Tcm preparation).tc.
73. (Tcm preparation).kw.

74. (Formula).sh.
75. (Formula).ti.
76. (Formula).ab.
77. (Formula).kf.
78. (Formula).tc.
79. (Formula).kw.
80. (Herbal prescription).sh.
81. (Herbal prescription).ti.
82. (Herbal prescription).ab.
83. (Herbal prescription).kf.
84. (Herbal prescription).tc.
85. (Herbal prescription).kw.
86. (Chinese herb compound).sh.
87. (Chinese herb compound).ti.
88. (Chinese herb compound).ab.
89. (Chinese herb compound).kf.
90. (Chinese herb compound).tc.
91. (Chinese herb compound).kw.
92. granular formaulation.ti.
93. granular formaulation.ab.
94. granular formaulation.kf.
95. granular formaulation.tc.
96. granular formaulation.kw.
97. granule.ti.
98. granule.ab.
99. granule.kf.
100. granule.tc.
101. granule.kw.
102. particle.ti.
103. particle.ab.
104. particle.kf.
105. particle.tc.
106. particle.kw.
107. keli.ti.
108. keli.ab.
109. keli.kf.
110. keli.tc.
111. keli.kw.
112. grain.ti.
113. grain.ab.
114. grain.kf.
115. grain.tc.
116. grain.kw.
117. or/14-116

118. 13 and 117

Search strategy for Embase

1. (HFMD).sh.
2. (HFMD).ti.
3. (HFMD).ab.
4. (HFMD).kw.
5. (Hand Foot and Mouth Disease).sh.
6. (Hand Foot and Mouth Disease).ti.
7. (Hand Foot and Mouth Disease).ab.
8. (Hand Foot and Mouth Disease).kw.
9. or/1-8
10. (Chinese drug therapy).sh.
11. (Chinese drug therapy).ti.
12. (Chinese drug therapy).ab.
13. (Chinese drug therapy).kw.
14. (Chinese herb Treatment).sh.
15. (Chinese herb Treatment).ti.
16. (Chinese herb Treatment).ab.
17. (Chinese herb Treatment).kw.
18. (Traditional Chinese medicin).sh.
19. (Traditional Chinese medicin).ti.
20. (Traditional Chinese medicin).ab.
21. (Traditional Chinese medicin).kw.
22. (Chinese medicine).sh.
23. (Chinese medicine).ti.
24. (Chinese medicine).ab.
25. (Chinese medicine).kw.
26. (Chinese patent drug).sh.
27. (Chinese patent drug).ti.
28. (Chinese patent drug).ab.
29. (Chinese patent drug).kw.
30. (Chinese patent medicine).sh.
31. (Chinese patent medicine).ti.
32. (Chinese patent medicine).ab.
33. (Chinese patent medicine).kw.
34. (Chinese traditional medicine).sh.
35. (Chinese traditional medicine).ti.
36. (Chinese traditional medicine).ab.
37. (Chinese traditional medicine).kw.
38. (Chinese preparation).sh.
39. (Chinese preparation).ti.
40. (Chinese preparation).ab.
41. (Chinese preparation).kw.

42. (Preparation Chinese materia medica).sh.
43. (Preparation Chinese materia medica).ti.
44. (Preparation Chinese materia medica).ab.
45. (Preparation Chinese materia medica).kw.
46. (Tcm preparation).sh.
47. (Tcm preparation).ti.
48. (Tcm preparation).ab.
49. (Tcm preparation).kw.
50. (Formula).sh.
51. (Formula).ti.
52. (Formula).ab.
53. (Formula).kw.
54. (Herbal prescription).sh.
55. (Herbal prescription).ti.
56. (Herbal prescription).ab.
57. (Herbal prescription).kw.
58. (Chinese herb compound).sh.
59. (Chinese herb compound).ti.
60. (Chinese herb compound).ab.
61. (Chinese herb compound).kw.
62. (granular formaulation).ti.
63. (granular formaulation).ab.
64. (granular formaulation).kw.
65. granule.ti.
66. granule.ab.
67. granule.kw.
68. particle.ti.
69. particle.ab.
70. particle.kw.
71. keli.ti.
72. keli.ab.
73. keli.kw.
74. grain.ti.
75. grain.ab.
76. grain.kw.
77. or/10-76
78. 9 and 77

Search strategy for Science direct

1. Title, abstract, keywords: ("HFMD" OR "Hand Foot and Mouth Disease" OR "Herpangina")
2. Title, abstract, keywords: ("Chinese drug therapy" OR "Chinese herb Treatment" OR "Traditional Chinese medicin" OR "Chinese medicine" OR "Chinese patent drug" OR "chinese patent medicine" OR "Chinese traditional medicine" OR "Chinese preparation"

OR “Preparation Chinese materia medica” OR “Tcm preparation” OR “formula” OR “herbal prescription” OR “Chinese herb compound” OR “granular formaulation” OR “granule” OR “particle” OR “keli” OR “grain”)

3. 1 and 2

Search strategy for Web of science

1. TS=(Hand Foot and Mouth Disease)
2. TI=(Hand Foot and Mouth Disease)
3. AB=(Hand Foot and Mouth Disease)
4. AK=(Hand Foot and Mouth Disease)
5. KP=(Hand Foot and Mouth Disease)
6. TS=HFMD
7. TI=HFMD
8. AB=HFMD
9. AK=HFMD
10. KP=HFMD
11. or/1-10
12. TS=(Chinese drug therapy)
13. TI=(Chinese drug therapy)
14. AB=(Chinese drug therapy)
15. AK=(Chinese drug therapy)
16. KP=(Chinese drug therapy)
17. TS=(Chinese herb Treatment)
18. TI=(Chinese herb Treatment)
19. AB=(Chinese herb Treatment)
20. AK=(Chinese herb Treatment)
21. KP=(Chinese herb Treatment)
22. TS=(Traditional Chinese medicin*)
23. TI=(Traditional Chinese medicin*)
24. AB=(Traditional Chinese medicin*)
25. AK=(Traditional Chinese medicin*)
26. KP=(Traditional Chinese medicin*)
27. TS=(Chinese medicine)
28. TI=(Chinese medicine)
29. AB=(Chinese medicine)
30. AK=(Chinese medicine)
31. KP=(Chinese medicine)
32. TS=(Chinese patent drug)
33. TI=(Chinese patent drug)
34. AB=(Chinese patent drug)
35. AK=(Chinese patent drug)
36. KP=(Chinese patent drug)
37. TS=(Chinese patent medicine)
38. TI=(Chinese patent medicine)

39. AB=(Chinese patent medicine)
40. AK=(Chinese patent medicine)
41. KP=(Chinese patent medicine)
42. TS=(Chinese traditional medicine)
43. TI=(Chinese traditional medicine)
44. AB=(Chinese traditional medicine)
45. AK=(Chinese traditional medicine)
46. KP=(Chinese traditional medicine)
47. TS=(Chinese preparation)
48. TI=(Chinese preparation)
49. AB=(Chinese preparation)
50. AK=(Chinese preparation)
51. KP=(Chinese preparation)
52. TS=(Preparation Chinese materia medica)
53. TI=(Preparation Chinese materia medica)
54. AB=(Preparation Chinese materia medica)
55. AK=(Preparation Chinese materia medica)
56. KP=(Preparation Chinese materia medica)
57. TS=(Tcm preparation)
58. TI=(Tcm preparation)
59. AB=(Tcm preparation)
60. AK=(Tcm preparation)
61. KP=(Tcm preparation)
62. TS=(Formula)
63. TI=(Formula)
64. AB=(Formula)
65. AK=(Formula)
66. KP=(Formula)
67. TS=(Herbal prescription)
68. TI=(Herbal prescription)
69. AB=(Herbal prescription)
70. AK=(Herbal prescription)
71. KP=(Herbal prescription)
72. TS=(Chinese herb compound)
73. TI=(Chinese herb compound)
74. AB=(Chinese herb compound)
75. AK=(Chinese herb compound)
76. KP=(Chinese herb compound)
77. TS=(Granular formaulation)
78. TI=(Granular formaulation)
79. AB=(Granular formaulation)
80. AK=(Granular formaulation)
81. KP=(Granular formaulation)
82. TS=(Granule)

83. TI=(Granule)
84. AB=(Granule)
85. AK=(Granule)
86. KP=(Granule)
87. TS=(Particle)
88. TI=(Particle)
89. AB=(Particle)
90. AK=(Particle)
91. KP=(Particle)
92. TS=(Keli)
93. TI=(Keli)
94. AB=(Keli)
95. AK=(Keli)
96. KP=(Keli)
97. TS=(Grain)
98. TI=(Grain)
99. AB=(Grain)
100. AK=(Grain)
101. KP=(Grain)
102. or/12-101
103. 11 and 102

Search strategy for China biology medicine disc (CBM)

1. "**shouzukoubing**"[mesh, exp]
2. "shouzukou"[Chinese title]
3. "shouzukou"[abstract]
4. "shouzukou"[key words]
5. "Hand % Foot % Mouth Disease"[English title]
6. "Hand % Foot % Mouth Disease"[abstract]
7. "Hand % Foot % Mouth Disease"[key words]
8. "HFMD"[English title]
9. "HFMD"[abstract]
10. "HFMD"[key words]
11. or/1-10
12. "**zhongyaoliaofa**"[mesh, exp]
13. "zhongyao"[Chinese title]
14. "zhongyao"[abstract]
15. "zhongyao"[key words]
16. "zhongcaoyao"[Chinese title]
17. "zhongcaoyao"[abstract]
18. "zhongcaoyao"[key words]
19. "zhongchengyao"[Chinese title]
20. "zhongchengyao"[abstract]
21. "zhongchengyao"[key words]

22. "chengfangzhiji"[Chinese title]
23. "chengfangzhiji"[abstract]
24. "chengfangzhiji"[key words]
25. "fangji"[Chinese title]
26. "fangji"[abstract]
27. "fangji"[key words]
28. "fufang"[Chinese title]
29. "fufang"[abstract]
30. "fufang"[key words]
31. "yaofang"[Chinese title]
32. "yaofang"[abstract]
33. "yaofang"[key words]
34. "zufang"[Chinese title]
35. "zufang"[abstract]
36. "zufang"[key words]
37. "chinese drug therapy"[Chinese title]
38. "chinese drug therapy"[abstract]
39. "chinese drug therapy"[key words]
40. "treatment with chinese herb"[Chinese title]
41. "treatment with chinese herb"[abstract]
42. "treatment with chinese herb"[key words]
43. "traditional chinese medicin%"[Chinese title]
44. "traditional chinese medicin%"[abstract]
45. "traditional chinese medicin%"[key words]
46. "chinese medicine"[Chinese title]
47. "chinese medicine"[abstract]
48. "chinese medicine"[key words]
49. "chinese patent %"[Chinese title]
50. "chinese patent %"[abstract]
51. "chinese patent %"[key words]
52. "chinese traditional % medicine"[Chinese title]
53. "chinese traditional % medicine"[abstract]
54. "chinese traditional % medicine"[key words]
55. "chinese % preparation"[Chinese title]
56. "chinese % preparation"[abstract]
57. "chinese % preparation"[key words]
58. "preparation of chinese materia medica"[Chinese title]
59. "preparation of chinese materia medica"[abstract]
60. "preparation of chinese materia medica"[key words]
61. "tcm preparation"[Chinese title]
62. "tcm preparation"[abstract]
63. "tcm preparation"[key words]
64. "formula"[Chinese title]
65. "formula"[abstract]

66. "formula"[key words]
67. "herbal prescription"[Chinese title]
68. "herbal prescription"[abstract]
69. "herbal prescription"[key words]
70. "chinese herb % compound"[Chinese title]
71. "chinese herb % compound"[abstract]
72. "chinese herb % compound"[key words]
73. "keli"[Chinese title]
74. "keli"[abstract]
75. "keli"[key words]
76. "chongji"[Chinese title]
77. "chongji"[abstract]
78. "chongji"[key words]
79. "granular formaulation"[English title]
80. "granular formaulation"[abstract]
81. "granular formaulation"[key words]
82. "granule"[English title]
83. "granule"[abstract]
84. "granule"[key words]
85. "particle"[English title]
86. "particle"[abstract]
87. "particle"[key words]
88. "keli"[English title]
89. "keli"[abstract]
90. "keli"[key words]
91. "grain"[English title]
92. "grain"[abstract]
93. "grain"[key words]
94. or/12-93
95. 11 and 94

Search strategy for China national knowledge infrastructure (CNKI)

1. SU=shouzukou
2. TI=shouzukou
3. KY=shouzukou
4. AB=shouzukou
5. SU=Hand*Foot*Mouth*Disease
6. TI=Hand*Foot*Mouth*Disease
7. KY=Hand*Foot*Mouth*Disease
8. AB=Hand*Foot*Mouth*Disease
9. or/1-8
10. SU=zhongyao
11. SU=zhongchengyao
12. SU=zhongcaoyao

13. SU=tianranyaowu
14. TI=zhongyao
15. TI=zhongchengyao
16. TI=zhongcaoyao
17. TI=tianranyaowu
18. KY=zhongyao
19. KY=zhongchengyao
20. KY=zhongcaoyao
21. KY=tianranyaowu
22. AB=zhongyao
23. AB=zhongchengyao
24. AB=zhongcaoyao
25. AB=tianranyaowu
26. SU=fangji
27. SU=chengfangzhiji
28. TI=fangji
29. TI=chengfangzhiji
30. KY=fangji
31. KY=chengfangzhiji
32. AB=fangji
33. AB=chengfangzhiji
34. SU=zufang
35. SU=yaofang
36. SU=fufang
37. TI=zufang
38. TI=yaofang
39. TI=fufang
40. KY=zufang
41. KY=yaofang
42. KY=fufang
43. AB=zufang
44. AB=yaofang
45. AB=fufang
46. SU=Chinese*drug*therapy
47. SU=Chinese*herb*Treatment
48. SU=Traditional*Chinese*medicin
49. SU=Chinese*medicine
50. TI=Chinese*drug*therapy
51. TI=Chinese*herb*Treatment
52. TI=Traditional*Chinese*medicin
53. TI=Chinese*medicine
54. KY=Chinese*drug*therapy
55. KY=Chinese*herb*Treatment
56. KY=Traditional*Chinese*medicin

57. KY=Chinese*medicine
58. AB=Chinese*drug*therapy
59. AB=Chinese*herb*Treatment
60. AB=Traditional*Chinese*medicin
61. AB=Chinese*medicine
62. SU=Chinese*patent*drug
63. SU=Chinese*traditional*medicine
64. TI=Chinese*patent*drug
65. TI=Chinese*traditional*medicine
66. KY=Chinese*patent*drug
67. KY=Chinese*traditional*medicine
68. AB=Chinese*patent*drug
69. AB=Chinese*traditional*medicine
70. SU=Chinese*preparation
71. SU=Tcm*preparation
72. SU=Preparation*Chinese*materia*medica
73. TI=Chinese*preparation
74. TI=Tcm*preparation
75. TI=Preparation*Chinese*materia*medica
76. KY=Chinese*preparation
77. KY=Tcm*preparation
78. KY=Preparation*Chinese*materia*medica
79. AB=Chinese*preparation
80. AB=Tcm*preparation
81. AB=Preparation*Chinese*materia*medica
82. SU=formula
83. SU=herbal*prescription
84. SU=Chinese*herb*compound
85. TI=formula
86. TI=herbal*prescription
87. TI=Chinese*herb*compound
88. KY=formula
89. KY=herbal*prescription
90. KY=Chinese*herb*compound
91. AB=formula
92. AB=herbal*prescription
93. AB=Chinese*herb*compound
94. SU=Chinese*patent*medicine
95. TI=Chinese*patent*medicine
96. KY=Chinese*patent*medicine
97. AB=Chinese*patent*medicine
98. SU=keli
99. TI=keli
100. KY=keli

101. AB=keli
102. SU=granular*formaulation
103. TI=granular*formaulation
104. KY=granular*formaulation
105. AB=granular*formaulation
106. SU=granule
107. TI=granule
108. KY=granule
109. AB=granule
110. SU=chongji
111. TI=chongji
112. KY=chongji
113. AB=chongji
114. SU=grain
115. TI=grain
116. KY=grain
117. AB=grain
118. SU=particle
119. TI=particle
120. KY=particle
121. AB=particle
122. SU=keli
123. TI=keli
124. KY=keli
125. AB=keli
126. or/10-125
127. 9 and 126

Search strategy for Wanfang database (Wanfang data)

Literature types: journal papers, academic papers and conference papers

1. [Mesh]shouzukou
2. [Title/Key words]shouzukou
3. [Abstract]shouzukou
4. [Mesh]Hand Foot and Mouth Disease
5. [Title/Key words]Hand Foot and Mouth Disease
6. [Abstract]Hand Foot and Mouth Disease
7. or/1-6
8. [Mesh]zhongyao
9. [Mesh]zhongcaoyao
10. [Mesh]zhongchengyao
11. [Mesh]tianranyaowu
12. [Title/Key words]zhongyao
13. [Title/Key words]zhongcaoyao
14. [Title/Key words]zhongchengyao

15. [Title/Key words]tianranyaowu
16. [Abstract]zhongyao
17. [Abstract]zhongcaoyao
18. [Abstract]zhongchengyao
19. [Abstract]tianranyaowu
20. [Mesh]fangji
21. [Mesh]chengfangzhiji
22. [Title/Key words]fangji
23. [Title/Key words]chengfangzhiji
24. [Abstract]fangji
25. [Abstract]chengfangzhiji
26. [Mesh]fufang
27. [Title/Key words]fufang
28. [Abstract]fufang
29. [Mesh]yaofang
30. [Title/Key words]yaofang
31. [Abstract]yaofang
32. [Mesh]zufang
33. [Title/Key words]zufang
34. [Abstract]zufang
35. [Mesh]Chinese drug therapy
36. [Title/Key words]Chinese drug therapy
37. [Abstract]Chinese drug therapy
38. [Mesh]Chinese herb Treatment
39. [Title/Key words]Chinese herb Treatment
40. [Abstract]Chinese herb Treatment
41. [Mesh]Traditional Chinese medicin
42. [Title/Key words]Traditional Chinese medicin
43. [Abstract]Traditional Chinese medicin
44. [Mesh]Chinese medicine
45. [Title/Key words]Chinese medicine
46. [Abstract]Chinese medicine
47. [Mesh]Chinese patent drug
48. [Title/Key words]Chinese patent drug
49. [Abstract]Chinese patent drug
50. [Mesh]chinese patent medicine
51. [Title/Key words]chinese patent medicine
52. [Abstract]chinese patent medicine
53. [Mesh]Chinese traditional medicine
54. [Title/Key words]Chinese traditional medicine
55. [Abstract]Chinese traditional medicine
56. [Mesh]Chinese preparation
57. [Title/Key words]Chinese preparation
58. [Abstract]Chinese preparation

59. [Mesh]Preparation Chinese materia medica
60. [Title/Key words]Preparation Chinese materia medica
61. [Abstract]Preparation Chinese materia medica
62. [Mesh]Tcm preparation
63. [Title/Key words]Tcm preparation
64. [Abstract]Tcm preparation
65. [Mesh]formula
66. [Title/Key words]formula
67. [Abstract]formula
68. [Mesh]herbal prescription
69. [Title/Key words]herbal prescription
70. [Abstract]herbal prescription
71. [Mesh]Chinese herb compound
72. [Title/Key words]Chinese herb compound
73. [Abstract]Chinese herb compound
74. [Mesh]keli
75. [Title/Key words]keli
76. [Abstract]keli
77. [Mesh]chongji
78. [Title/Key words]chongji
79. [Abstract]chongji
80. [Mesh]granular formaulation
81. [Title/Key words]granular formaulation
82. [Abstract]granular formaulation
83. [Mesh]granule
84. [Title/Key words]granule
85. [Abstract]granule
86. [Mesh]particle
87. [Title/Key words]particle
88. [Abstract]particle
89. [Mesh]keli
90. [Title/Key words]keli
91. [Abstract]keli
92. [Mesh]grain
93. [Title/Key words]grain
94. [Abstract]grain
95. or/8-94
96. 7 and 95

Search strategy for China science and technology journal database (VIP)

1. M=(shouzukoubing)
2. M=(Hand Foot Mouth Disease)
3. M=(Herpangina)
4. R=(shouzukoubing)
5. R=(Hand Foot Mouth Disease)

6. or/1-5
7. M=(zhongyao)
8. R=(zhongyao)
9. M=(zhongcaoyao)
10. R=(zhongcaoyao)
11. M=(tianranyaowu)
12. R=(tianranyaowu)
13. M=(zhongchengyao)
14. R=(zhongchengyao)
15. M=(chengfangzhiji)
16. R=(chengfangzhiji)
17. M=(fangji)
18. R=(fangji)
19. M=(fufang)
20. R=(fufang)
21. M=(yaofang)
22. R=(yaofang)
23. M=(zufang)
24. R=(zufang)
25. M=(Chinese drug therapy)
26. R=(Chinese drug therapy)
27. M=(Chinese herb Treatment)
28. R=(Chinese herb Treatment)
29. M=(Traditional Chinese medicin)
30. R=(Traditional Chinese medicin)
31. M=(Chinese medicine)
32. R=(Chinese medicine)
33. M=(Chinese patent drug)
34. R=(Chinese patent drug)
35. M=(chinese patent medicine)
36. R=(chinese patent medicine)
37. M=(Chinese traditional medicine)
38. R=(Chinese traditional medicine)
39. M=(Chinese preparation)
40. R=(Chinese preparation)
41. M=(Preparation Chinese materia medica)
42. R=(Preparation Chinese materia medica)
43. M=(Tcm preparation)
44. R=(Tcm preparation)
45. M=(formula)
46. R=(formula)
47. M=(herbal prescription)
48. R=(herbal prescription)
49. M=(Chinese herb compound)

50. R=(Chinese herb compound)
51. M=(keli)
52. R=(keli)
53. M=(granular formaulation)
54. R=(granular formaulation)
55. M=(granule)
56. R=(granule)
57. M=(chongji)
58. R=(chongji)
59. M=(grain)
60. R=(grain)
61. M=(particle)
62. R=(particle)
63. M=(keli)
64. R=(keli)
65. or/7-64
66. 8 and 65

Clinical trial registry

Search “Hand Foot Mouth Disease” or “HFMD”

Chinese clinical trial registry

Search “shouzukou”

Appendix S2

References for included studies

[1] Wang Z Y, Huang J Q. Clinical observation of Lianhua Qingwen granule combined with ribavirin in the treatment of hand, foot and mouth disease in children[J]. Chin Med J Metall Indus (Chin.). 2021, 38(01): 25-26.
[2] Liu H. The effect of Qingkailing granule combined with ribavirin in the treatment of hand, foot and mouth disease in children[J]. Contemporary Medical Symposium (Chin.). 2021, 19: 92-93.
[3] Dai Y Q. Effectiveness Analysis of Xiao'er Chiqiao Qingre Granules Combined with Ribavirin in the Treatment of HFMD[J]. Guide Chin. Med. (Chin.). 2020, 18(6): 224-225.
[4] Di J H. Effectiveness Analysis of Pediatric Resuqing Granule in Hand, Foot and Mouth Disease [J]. System Medicine (Chin.). 2020, 5(06): 90-92.
[5] Yang W. Study on the Superiority of Ribavirin and Jinlianhua Granules in Children with HFMD[J]. Food Science (Chin.). 2020, 0(1).
[6] Zhao X R, Luo H Q. Efficacy of Kanggan Granule Combined with Ｒibavirin in the Treatment of Hand Foot Mouth Disease in Children and its Effect on Immune Function and Inflammatory Factor[J]. Evaluation and Analysis of Drug-Use in Hospitals of China (Chin.). 2020, 20(10): 1168-1170.
[7] Yu J, Fu Q Q. Clinical Study on Jinlianhua Granules Combined with Ribavirin in Treatment of Hand Foot and Mouth Disease in Children[J]. Drugs & Clinic (Chin.). 2019, 34(4): 1050-1054.
[8] Li H P, Huang A L, Hu X J. Clinical Observation on Effectiveness of Xiao'er Chiqiao Qingre Granules in the Treatment of HFMD in Children[J]. Harbin Med J (Chin.). 2019, 39(3): 270-271.
[9] Yan Z J, Gong J. Observation on Effectiveness of Xiao'er Chiqiao Qingre Granules in the Treatment of HFMD[J]. Journal of Clinical Medical (Chin.). 2019, 6(03): 170-172.
[10] Cai X C. Efficacy and Safety of Xiao'er Jinqiao Granules Combined with Ribavirin in the Treatment of HFMD[J]. YIYAOJIE (Chin.). 2019, 0(5): 21.
[11] Hu Z X. Effectiveness Analysis of Ribavirin Injection plus Kouyanqing Granules in the Treatment of HFMD in Children[J]. Contemporary Medical Symposium (Chin.). 2019, 17(8): 156-157.
[12] Li Y X. Clinical Observation on Effectiveness of TCM Combined with Western Medicine in the Treatment of HFMD[J]. J Pract Tradit Chin Med (Chin.). 2019, 35(10): 1216-1217.
[13] Ye J Y, Yuan H Q, Wang M. Clinical Observation on Shanla Meiye Granules Combined with Western Medicine for Hand-Foot-Mouth Disease in Children[J]. Journal of New Chinese Medicine (Chin.). 2018, 50(11): 147-149.
[14] Yang Y. Clinical Observation of Xiao' er Chiqiao Qingre Granules Combined with Western Medicine for Hand-Foot-Mouth Disease[J]. Journal of New Chinese Medicine (Chin.). 2018, 50(10): 127-129.
[15] Zeng Y. Effect of ribavirin combined with yanning granule in treatment of hand, foot and mouth disease in children[J]. Contemporary Medical Symposium (Chin.). 2018, 16(23): 109-110.
[16] He Y F. Effectiveness of Ertong Huichun Granules in the Treatment of HFMD in Children[J]. Heilongjiang Med (Chin.). 2017, 40(5): 146-147.
[17] Han C Y. Clinical Observation on 60 Cases of Lianhua Qingwen Granules Combined with Ribavirin in the Treatment of HFMD in Children[J]. Chinese Journal of Ethnomedicine and Ethnopharmacy (Chin.). 2017, 26(11): 108-110.
[18] Yu S J. Analysis of Children's Hand-foot-mouth Disease Treated by Children's Soybean

Granule Combined with Ribavirin Aerosol[J]. Chin Pediatr of Integr Tradit West Med (Chin.). 2017, 9(1): 67-69.
[19] Zhang J H. Effectiveness Analysis of Xiao'er Chiqiao Qingre Granules in the Treatment of HFMD in Children[J]. Chinese and Foreign Medical Research (Chin.). 2017, 15(20): 116-117.
[20] Zheng G L, Lv S H, Kang F. Clinical Observation of Resuqing Granules for Children Combined with Ribavirin in the Treatment of Hand-foot-mouth Diseases[J]. China Modern Medicine (Chin.). 2017, 24(28): 132-134.
[21] Ke W, Li Y. Observation on Effectiveness of Jinyexiudu granule in children with hand, foot and mouth disease[J]. Lishizhen Medicine and Materia Medica Research (Chin.). 2017, 28(4): 916-917.
[22] Wang Z H. Clinical Observation on Effectiveness of Xiao'er Chaigui Ture Granules Combined with Ribavirin Granules in the Treatment of HFMD in Children[J]. China Prac Med (Chin.). 2016, 11(23): 135-136.
[23] Ma L Y, Li Y H. Clinical Study on Xiao'er Chiqiao Qingre Granules Combined with Ribavirin Aerosol in the Treatment of HFMD in Children[J]. For all Health (Chin.). 2016, 10(4): 156-157.
[24] Wu X Q, Sun D F, Feng J J. Observation on Effectiveness of Xiao'er Chiqiao Qingre Granules as Adjuvant Therapy in the Treatment of HFMD in Children[J]. Journal of Chinese Medicinal Materials (Chin.). 2016, 39(10): 2376-2378.
[25] Sun L, Xiang Y. Observation on Effectiveness of Jinlianhua Granules in the Treatment of HFMD in Children[J]. Journal of New Chinese Medicine (Chin.). 2016, 48(4): 140-141.
[26] Wang J J, Yang L P. Observation on Effectiveness of Lianhua Qingwen Granules Combined with Ribavirin Aerosol in the Treatment of HFMD in Children[J]. Chin J of Clinical Rational Drug Use (Chin.). 2015, 8(17): 67-68.
[27] Zheng Y F, Wu Q Y. Effect Observation of 28 Cases of Hand-foot-mouth Disease Treated with Xiaoer Chiqiao Qingre Keli and Western Medicine[J]. J. Pediatrics of TCM (Chin.). 2015, 11(1): 35-37.
[28] Luo L S. Clinical Observation on Effectiveness of TCM Combined with Western Medicine in the Treatment of mild HFMD[J]. World Latest Medicine Information (Chin.). 2015, 15(51): 151-152.
[29] Liu X L, Tian W Q. Observation on Effectiveness of Lianhua Qingwen Granules in the Treatment of HFMD[J]. Xiandai Yangsheng B (Chin.). 2014(3): 164.
[30] Shen W L, Xiang S Y, Zhao C P. Clinical Observation on Treating 55 Cases of HFMD with Qinkai Ling Granule plus Ribavirin Injection[J]. Clinical Journal of Chinese Medicine (Chin.). 2014, 6(7): 94-95.
[31] Xu X J. Clinical Observation on Effectiveness of TCM Combined with Western Medicine in the Treatment of HFMD[J]. Journal of New Chinese Medicine (Chin.). 2014, 46(12): 147-148.
[32] Yu H H, Lv M Z. Observation on Effectiveness of Fangfeng Tongsheng Granules Combined with Ribavirin in the Treatment of HFMD in Children[J]. Zhejiang JITCWM (Chin.). 2013, 23(9): 739-740.
[33] He W, Zheng B, Li X Y, et al. Observation on Effectiveness of Lianhua Qingwen Granules in the Treatment of HFMD of EV71[J]. Zhejiang JITCWM (Chin.). 2013, 23(2): 152-153.
[34] Zhong X D, Luo Y F. Clinical Observation on 120 Cases of Qingkailing Granules as Adjuvant Therapy in the Treatment of HFMD in Children[J]. Chinese Community Doctors·Medicine (Chin.). 2013, 15(10): 205.

[35] Xu J. Observation on Effectiveness of Xiaoer Chaigui Ture Granules Combined with Ribavirin in the Treatment of HFMD[J]. Zhejiang JITCWM (Chin.). 2013, 23(5): 405-406.
[36] Wu Q. Analysis of Therapeutic Effect of Bairui Particles for Hand-foot-mouth Disease[J]. Chinese Journal of Experimental Traditional Medical Formulae (Chin.). 2012, 18(13): 276-278.
[37] Guo L F. Effect of Ertong Huichun Granules in the Treatment of HFMD in Children[J]. Pract Clin Med (Chin.). 2012, 13(4): 79, 89.
[38] Zhang Y. Observation on Effectiveness of Reduping Granules Combined with Ribavirin in the Treatment of HFMD[J]. Jining Med Univ (Chin.). 2012, 35(4): 268-269.
[39] Dai Y H. Effect of Shanlameiye Granules in the Treatment of HFMD[J]. Chinese Medicine Modern Distance Education of China (Chin.). 2012, 10(5): 17-18.
[40] Tang H. Observation on Effectiveness of Xiao'er Chiqiao Qingre Granules Combined with Ribavirin Aerosol in the Treatment of HFMD in Children[J]. Modern Journal of Integrated Traditional Chinese and Western Medicine (Chin.). 2012, 21(2): 175, 178.
[41] Li J, Kong W Z. Clinical Observation on 50 Cases of Kanggan Granules in the Treatment HFMD[J]. Journal of Frontiers of Medicine (Chin.). 2012(35): 137.
[42] Huang Q L, Wu Y, Wang X, et al. Clinical Observation on 45 Cases of Houerhuan Xiaoyan Granules Combined with Ribavirin Aerosol in the Treatment of Phase Ⅰ HFMD[J]. Zhejiang Journal of Traditional Chinese Medicine (Chin.). 2011, 46(2): 126.
[43] Cai S Y, Yan Z G, Yang X H. Clinical observation on treating hand, foot and mouth disease by using Lianhuaqingwen granules combined with Ribavirin[J]. J Med Forum (Chin.). 2011, 32(16): 76-78.
[44] Yang J W. Comparison on Effectiveness between two therapeutic treatments of HFMD in Children[J]. Guide Chin. Med. (Chin.). 2011, 9(9): 71-72.
[45] Guo X Y, Xu Y, Fang J Q. Observation on 80 Cases of Qingkailing Granules as Adjuvant Therapy in the Treatment of HFMD[J]. JETCM (Chin.). 2009, 18(9): 1449-1460.

Appendix S3

Characteristics of included studies

Table S3. Characteristics of the included studies

<table>
<tr><th rowspan="2">NO.</th><th rowspan="2">Study</th><th colspan="2">Age/
$\bar{X}$± S (R), y</th><th rowspan="2">Gender M/F</th><th colspan="2">Course/
$\bar{X}$± S (R), d</th><th rowspan="2">Sample size (C/T)</th><th rowspan="2">Conventional treatment</th><th rowspan="2">C</th><th colspan="2">T</th><th rowspan="2">Duration/d</th><th rowspan="2">Outcomes</th></tr>
<tr><th>C</th><th>T</th><th>C</th><th>T</th><th>Drug</th><th>Dose</th></tr>
<tr><td rowspan="2">1</td><td rowspan="2">Wu, 2012</td><td colspan="2">3.21±1.20
(0.92~7.50)</td><td rowspan="2">51/49</td><td colspan="2" rowspan="2">NA</td><td rowspan="2">50/50</td><td rowspan="2">Ribavirin injection</td><td rowspan="2">-</td><td rowspan="2">BRGs</td><td rowspan="2">2.5g(<3year), 5g(>3years); tid</td><td rowspan="2">5</td><td rowspan="2">①②③⑥⑦</td></tr>
<tr><td>3.19±1.20
(1.00~7.50)</td><td>3.213±1.21
(0.92~7.00)</td></tr>
<tr><td rowspan="2">2</td><td rowspan="2">He, 2017</td><td colspan="2">(0.417~6.8)</td><td rowspan="2">51/47</td><td colspan="2">0.66±0.3
(0.49~0.91)</td><td rowspan="2">49/49</td><td rowspan="2">Ribavirin</td><td rowspan="2">-</td><td rowspan="2">EHGs</td><td rowspan="2">0.25g(<1year),0.5g(1~2years),0.6g(2~4years),1g(4~7years); tid</td><td rowspan="2">7</td><td rowspan="2">①②③④⑧</td></tr>
<tr><td>n=13 (0.458~1); n=24 (1~3); n=12 (3~6.8)</td><td>n=12 (0.417~1); n=23 (1~3); n=14 (3~6.8)</td><td>62/64</td><td>0.64±0.28
(0.55~0.91)</td></tr>
<tr><td rowspan="2">3</td><td rowspan="2">Guo, 2012</td><td colspan="2">(0.5~6)</td><td rowspan="2">73/53</td><td colspan="2" rowspan="2">(0~7)
45/45</td><td rowspan="2">62/64</td><td rowspan="2">Ribavirin injection</td><td rowspan="2">-</td><td rowspan="2">EHGs</td><td rowspan="2">0.25g(<1year),0.5g(≥1~2years),0.6g(>2~4years),1g(>4~7years); bid~tid</td><td rowspan="2">7</td><td rowspan="2">①③⑥</td></tr>
<tr><td>n=24 (<1); n=35 (≥1~3); n=3 (≥4~6)</td><td>n=27 (<1); n=34 (≥1~3); n=3 (≥4~6)</td></tr>
<tr><td rowspan="2">4</td><td rowspan="2">Yu and Lv, 2013</td><td colspan="2">2.55±0.6
(3~6)</td><td rowspan="2">45/58</td><td colspan="2">1.14±0.32</td><td rowspan="2">47/56</td><td rowspan="2">Ribavirin Granules</td><td rowspan="2">-</td><td rowspan="2">FTGs</td><td rowspan="2">3g; bid</td><td rowspan="2">5</td><td rowspan="2">①③⑤⑦</td></tr>
<tr><td>2.5±0.7</td><td>2.6±0.5</td><td>49/54</td><td>1.18±0.34</td></tr>
<tr><td rowspan="2">5</td><td rowspan="2">Huang et al., 2011</td><td colspan="2">2.45±0.52</td><td rowspan="2">46/44</td><td colspan="2" rowspan="2">NA</td><td rowspan="2">45/45</td><td rowspan="2">Ribavirin Aerosol</td><td rowspan="2">-</td><td rowspan="2">HXGs</td><td rowspan="2">2g(<1year), tid;3g(1~3years), tid;6g(3~6years), bid;6g(>6years), tid</td><td rowspan="2">5</td><td rowspan="2">①③④⑥⑧</td></tr>
<tr><td>2.30±0.50</td><td>2.60±0.50</td></tr>
<tr><td rowspan="2">6</td><td rowspan="2">Ke and Li, 2017</td><td colspan="2">2.75±0.05
(0.5~5)</td><td rowspan="2">145/95</td><td colspan="2" rowspan="2">NA</td><td rowspan="2">120/120</td><td rowspan="2">Ribavirin injection</td><td rowspan="2">-</td><td rowspan="2">JBGs</td><td rowspan="2">NA</td><td rowspan="2">3-6</td><td rowspan="2">③</td></tr>
<tr><td>2.80</td><td>2.70</td></tr>
<tr><td>7</td><td>Yang, 2020</td><td colspan="2">3.25±0.76
(1~6)</td><td>71/61</td><td colspan="2">5.37±2.12
(1~8)</td><td>66/66</td><td>Ribavirin Granules</td><td>-</td><td>JLHGs</td><td>8g(1~3years), 16g(>3years); tid</td><td>7</td><td>①②③④⑤⑦</td></tr>
<tr><td rowspan="2">8</td><td rowspan="2">Yu and Fu, 2019</td><td colspan="2">2.96±0.57
(1.17~3.58)</td><td rowspan="2">69/39</td><td colspan="2" rowspan="2">5.40±2.30
(1~9)</td><td rowspan="2">54/54</td><td rowspan="2">Ribavirin Granules</td><td rowspan="2">-</td><td rowspan="2">JLHGs</td><td rowspan="2">8g(1~3years), 16g(>3years); tid</td><td rowspan="2">7</td><td rowspan="2">①②③④⑤⑦⑧</td></tr>
<tr><td>2.93±0.53
(1.25~3.58)</td><td>2.93±0.54
(1.17~3.58)</td></tr>
<tr><td rowspan="2">9</td><td rowspan="2">Sun and Xiang, 2016</td><td colspan="2">2.60±0.27
(1.00~7.00)</td><td rowspan="2">31/29</td><td colspan="2">2.40±0.27
(0.50~4.00)</td><td rowspan="2">30/30</td><td rowspan="2">Ribavirin</td><td rowspan="2">-</td><td rowspan="2">JLHGs</td><td rowspan="2">8g(1~3years), 16g(4~7years); tid</td><td rowspan="2">7</td><td rowspan="2">①②③④⑤</td></tr>
<tr><td>2.70±0.20
(1.00~7.00)</td><td>2.50±0.30
(1.00~6.00)</td><td>2.50±0.20
(0.5~4.00)</td><td>2.30±0.30
(1.00~4.00)</td></tr>
<tr><td rowspan="2">10</td><td rowspan="2">Zhao and Luo, 2020</td><td colspan="2">3.31±0.29</td><td rowspan="2">72/50</td><td colspan="2">2.91±0.29</td><td rowspan="2">61/61</td><td rowspan="2">Ribavirin tablet</td><td rowspan="2">-</td><td rowspan="2">KangGs</td><td rowspan="2">10g; tid</td><td rowspan="2">5-7</td><td rowspan="2">①②③④⑤⑦</td></tr>
<tr><td>3.28±0.25</td><td>3.33±0.23</td><td>2.89±0.30</td><td>2.92±0.28</td></tr>
</table>

11	Li and Kong, 2012	(0.50~3)			45/51	NA			46/50	Ribavirin	-	KangGs	NA; tid	3	②③⑤⑧
12	Yang, 2011	2.82±0.56 (0.75~7.00)			187/156	2.66±2.64 (1.00~5.00)			180/163	Ribavirin Granules	-	KangGs	5g; tid	7	①③⑥⑦
13	Hu, 2019	(3.20~0.75)	(3.50~7.4)	(3.20~7.5)	80/80	NA			80/80	Ribavirin injection	-	KouGs	6g; qd ~ bid	5	①②③④⑦
14	Xu, 2014	5.30±0.45 (0.50~10)	4.2 (0.50~8)	5.1 (0.75~10)	149/103	(0.25~2)			122/130	Ribavirin Granules	-	KouGs	1.5g(<5years) ,3g(>5years); bid	2-3	①③⑥⑦
15	Wang and Huang, 2021	(1~12)	(2~12)	(1~11)	62/58	(6~13)	(7~13)	(6~12)	60/60	Ribavirin injection	-	LQGs	2g(1~3years), 3g(3~12years); tid	7	①②③④⑥
16	Han, 2017	2.75±1.64 (1~4)	2.7±1.7 (1~5)	2.8±1.6 (1~4)	67/53	1.25±0.55 (1~4)	43/43	1.3±0.5 (1~3)	60/60	Ribavirin Aerosol	-	LQGs	2/3g(1~3year) ,1g(4~5years) ; tid	5	①②③④⑦
17	Wang and Yang, 2015	2.58±0.35	2.55±0.38	2.61±0.33	56/47	NA 50/50			49/54	Ribavirin Aerosol	-	LQGs	2g(1~3years), 3g(4~6years), 6g(>6years); tid	3-7	②③⑥
18	Liu and Tian, 2014	3.2 (1~5)			45/41	NA			63/63	Ribavirin Granules + water sobale vitamin injection	-	LQGs	1.5g(1~≤2yea rs),3g(2~≤5ye ars); tid	7	①③④
19	Cai et al., 2011	2.39±0.27	2.39±0.31	2.4±0.23	44/23	NA 84/84			36/31	Ribavirin Granules + water sobale vitamin injection	-	LQGs	1.5g(1~≤2yea rs),3g(2~≤5ye ars); tid	7	②③⑥⑧
20	He et al., 2013	2.36±0.21	2.35±0.2	2.36±0.23	78/42	3.74±0.64	31/31	3.71±0.65	60/60	Ribavirin Injection + Vit C Injection	-	LQGs	3g(≤3years),6 g(>3years); tid	7	①③⑤⑥
21	Liu, 2021	3.67±1.48	3.71±1.47	3.63±1.50	51/35	3.69±0.87	3.52±0.90	3.86±0.82	43/43	Ribavirin tablet	-	QGs	200mg/(kg·d)	7	③④⑤⑥
22	Shen et al., 2014	2.65±1.15 (0.67~6.70)	232.8 (0.75~6.60)	2.5 (0.67~6.70)	57/53	2.55±0.05 (1~3)	2.6 (1~3)	2.5 (1~3)	55/55	Ribavirin injection	-	QGs	5~10g; bid~tid	7	①②③④⑦
23	Zhong and Luo, 2013		n=72 (1~4); n=28 (>4)	n=84 (1~4); n=36 (>4)	118/102	NA			100/120	Ribavirin	-	QGs	1~1.5g(0.5~1 year),1.5~2g(1~3years),2~3 g(4~6years); bid	5-7	①③⑥⑧

24	Guo et al., 2009	n=122 (<3); n=23 (3~5); n=7 (>5)			78/74	NA			72/80	Ribavirin	-	QGs	5g(<1year), bid;5g(1~3years), tid;20/3g(3~5 years), tid;10g(>5years), tid	5	①③④⑤⑥
25	Zhang, 2012	(0.5~8)			72/54	(1~2)			60/66	Ribavirin injection	-	RGs	7/3g(<1year), tid;3.5g(1~3years), tid;7g(3~8years), bid	6	①③⑥
26	Ye et al., 2018	5.51±2.28 (0.583~8)	5.38±2.35 (0.583~8)	5.63±2.22 (0.667~8)	55/31	3.16±1.53 (1~5)	53/53	3.13±1.56 (1~5)	43/43	Ribavirin Granules + Montmorillonite Powder	-	SGs	10/3g(<1year),5g(1~4years),10g(>4years); tid	7	①⑧
27	Dai, 2012	(0.5~12)			54/46	NA			50/50	Ribavirin Granules	-	SGs	10/3g(<1year),5g(1~3years),10g(>3years); tid	5	①②③⑥⑧
28	Wang, 2016	3.65±0.8 (0.417~6)	3.7±0.8 (0.5~6)	3.6±0.8 (0.417~6)	69/59	NA<br>54/54			46/64	Ribavirin Granules	-	XCTGs	2.5g(<1year), 5g(1~3years), 7.5g(4~6years); qid	3	①③⑥⑦
29	Xu, 2013	3.61±0.27 (0.417~5)	3.5±0.3 (0.417~5)	3.7±0.2 (0.417~5)	43/39	NA<br>65/76			37/45	Ribavirin Injection	-	XCTGs	2.5g(<1year), 5g(~3years),7.5g(~6years); tid	5	①③
30	Dai, 2020	2.91±0.67 (0.5~5.5)	2.87±0.66 (0.667~5)	2.95±0.69 (0.5~5.5)	101/67	NA<br>18/24			84/84	Ribavirin Injection	-	XCQGs	1~2g(<1year), 2~3g(1~3years),3~4g(4~6years); tid	2~7	①③④
31	Li et al., 2019	2.14±0.44 (0~5)	2.14±0.43 (0~5)	2.13±0.45 (0~5)	NA	2.13±0.59 (1~6)	62/64	2.07±0.61 (1~5)	31/31	Ribavirin + Vit C Injection	-	XCQGs	1g(<1year),2g (1~4years),4g (>4years); tid	3~7	①③⑥⑦
32	Yan and Gong, 2019	2.8±1.4 (0.6~5)			200/180	2.05±1.21 (0.4~3.7)			190/190	Ribavirin Aerosol + Vit C & Vit B6 Injection	-	XCQGs	1g(0.6~1year),2g(1~3years),3g(>3years); tid	3~7	①③⑥⑦⑧
33	Li, 2019	1.64±0.42	1.62±0.41	1.65±0.44	59/47	3.1±1.34	60/60	3.08±1.24	53/53	Ribavirin Injection	-	XCQGs	1~2g(<1year), 2~3g(1~2years); tid	3~5	①③⑥

34	Yang, 2018	3.7±1.15 (2~6)	3.6±1.1 (2~6)	3.8±1.2 (2~6)	69/49	3.85±0.65 (1~8)	43/43	3.9±0.6 (1~8)	59/59	Ribavirin Granules + Vit C Injection + Cefuroxime sodium Injection	-	XCQGs	2~3g(2~3year),3~4g(4~7years); tid	7	①②③⑥⑦⑧
35	Yu, 2017	2.2±0.51 (0~4)	2.2±0.4 (0~3.8)	2.2±0.6 (0~4)	49/39	NA 60/60			39/39	Ribavirin Aerosol	-	XCQGs	1~2g(0.5~1year),2~3g(>1~3years),3~4g(>3~6years); tid	7	①②③⑥⑧
36	Zhang, 2017	2.5 (0.5~5)			76/70	2.3 (0.5~3.6)			67/79	Ribavirin Aerosol + Vit C & Vit B6	-	XCQGs	1g(0.5~1year),2g(1~3years),3g(>3years); tid	7	①②③⑤⑥⑦
37	Ma and Li, 2016	4.45±1.35 (0.333~8)	4.6±1.4 (0.333~8)	4.3±1.3 (0.417~8)	40/40	1.45±0.49 (1~4)	50/50	1.4±0.54 (1~3)	40/40	Ribavirin Aerosol	-	XCQGs	1~2g(0.5~1year),2~3g(1~3years),3~4g(4~6years),4~5g(7~9years); tid	5	①③⑥⑦⑧
38	Wu et al., 2016	3.2±1.5 (0.5~5)	3.3±1.6 (0.5~5)	3.1±1.4 (0.5~5)	61/47	3±1.45 (1~5)	37/45	2.9±1.4 (1~5)	54/54	Ribavirin + Vit C	-	XCQGs	1g(<1year),2g(1~4years),4g(>4years); tid	5	①②③⑥⑦⑧
39	Luo, 2015	2.8 (0.5~8)			60/34	NA			47/47	Ribavirin Aerosol	-	XCQGs	1~2g(0.5~1year),2~3g(1~3years),3~4g(4~6years),4~5g(7~9years),6g(>10years); tid	3-5	①②③⑥⑧
40	Zheng and Wu, 2015	2.24±1.27	2.12±1.33	2.35±1.23	25/31	(3~5)	22/22	(3~5)	28/28	Ribavirin Aerosol	-	XCQGs	1~2g(0.5~1year),2~3g(1~3years),3~4g(4~6years); tid~qid	7	①③⑥
41	Tang, 2012	2.75±0.5	2.7±0.53	2.8±0.51	60/34	NA			47/47	Ribavirin Aerosol	-	XCQGs	1~2g(0.5~1year),2~3g(1~3years),3~4g(4~6years),4~5g(7~9years),6g(>10years); tid	5	①②③⑥⑧
42	Cai, 2019	4.29±0.21 (2~6)	4.36±0.24 (3~6)	4.22±0.16 (2~6)	45/42	3.03±0.22 (1~5)	3.1±0.07 (2~5)	2.96±0.28 (1~5)	43/44	Ribavirin Injection	-	XJGs	7.5g(5~7years), tid; 7.5g(8~10years),qid; 10g(11~14years), tid	5	①②③⑥

<table>
<tr><td rowspan="2">43</td><td rowspan="2">Di, 2020</td><td colspan="2">3.20±0.60</td><td rowspan="2">61/67</td><td colspan="2">1.30±0.40</td><td rowspan="2">64/64</td><td rowspan="2">Ribavirin Injection</td><td rowspan="2">-</td><td rowspan="2">XRGs</td><td rowspan="2">0.5~1g(<1year),1~2g(1~3years); tid~qid</td><td rowspan="2">7</td><td rowspan="2">①③⑥⑧</td></tr>
<tr><td>3.20±0.50</td><td>3.10±0.60</td><td>1.20±0.40</td><td>1.30±0.30</td></tr>
<tr><td rowspan="2">44</td><td rowspan="2">Zheng et al., 2017</td><td colspan="2">2.7±0.4 (0.75~6)</td><td rowspan="2">54/26</td><td rowspan="2" colspan="2">NA</td><td rowspan="2">40/40</td><td rowspan="2">Ribavirin Injection</td><td rowspan="2">-</td><td rowspan="2">XRGs</td><td rowspan="2">2g; bid</td><td rowspan="2">7</td><td rowspan="2">①③⑥⑧</td></tr>
<tr><td>2.65±0.48 (0.75~5)</td><td>2.73±0.38 (0.83~6)</td></tr>
<tr><td>45</td><td>Zeng, 2018</td><td colspan="2">(3~7)</td><td>35/30</td><td colspan="2">NA</td><td>30/35</td><td>Ribavirin Injection</td><td>-</td><td>YNGs</td><td>14g; tid~qid</td><td>3-5</td><td>①②③④⑦</td></tr>
</table>

Note: $\overline{X}$± S (R), mean ± standard deviation (range); y, year; d, day; C, Control Group; T, Test Group; "-" denotes "No medication"; NA, Not Available; BRGs, Bairui Granules; EHGs, Ertong Huichun Granules; FTGs, Fangfeng Tongsheng Granules; HXGs, Houerhuan Xiaoyan Granules; JLHGs, Jinlianhua Granules; JBGs, Jinye Baidu Granules; KangGs, Kanggan Granules; KouGs, Kouyanqing Granules; LQGs, Lianhua Qingwen Granules; QGs, Qingkailing Granules; RGs, Reduping Granules; SGs, Shanlameiye Granules; XCTGs, Xiao'er Chaigui Ture Granules; XCQGs, Xiao'er Chiqiao Qingre Granules; XJGs, Xiao'er Jinqiao Granules; XRGs, Xiao'er Resuqing Granules; YNGs, Yanning Granules; ①, Total effectiveness rate (TER); ②, Adverse effect rate (ADR); ③, Fever Clearance Time; ④, Disappearance/Scabbing time of vesicles; ⑤, Improvement time in appetite; ⑥, Disappearance/scabbing time of rash; ⑦, Disappearance/healing time of ulcers; ⑧, Hospitalization/healing/treatment time.

Appendix S4

Baseline characteristics of age and course among all studies

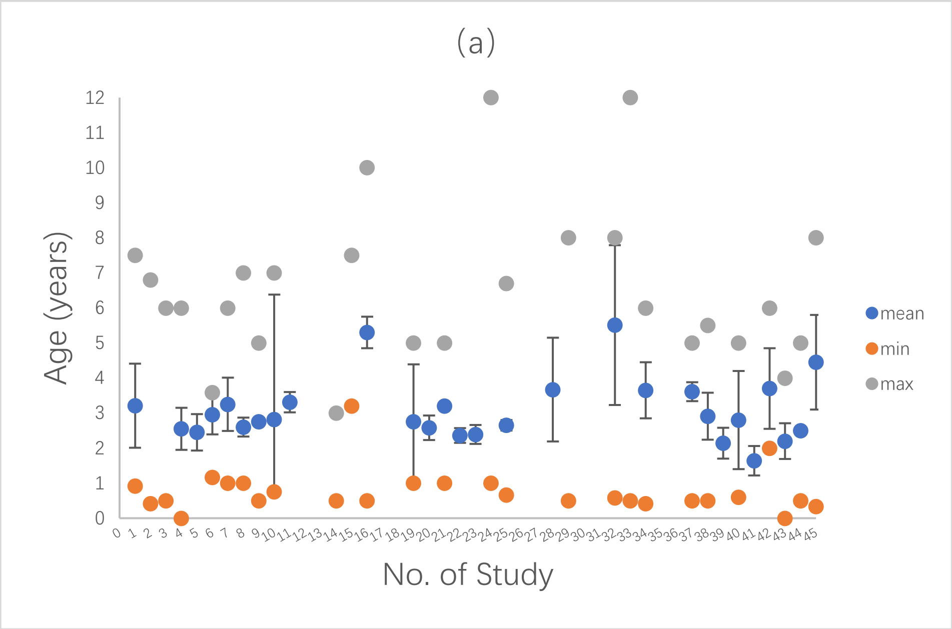

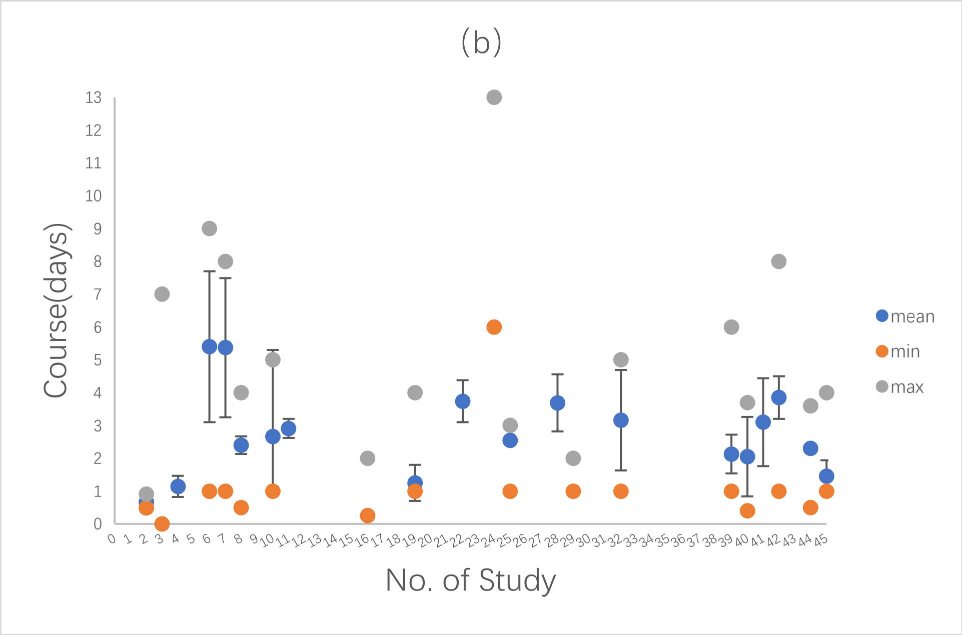

Figure S4. Characteristics of age and course among all studies on mild HFMD.

Appendix S5

Information of HGs-HD

Table S5 Information of HGs-HD

HGs-HD	Source	Species / Raw materials	Botanical plant names or chemical formula	Therapeutic claims in TCM	Quality control reported? (Y/N)
Bairui Granules (BRGs) △	Anhui Jiuhua Huayuan Pharmaceutical Co., Ltd	1. Herba Thes11	1. *Thesium chinense* Turcz.	**clear heat** and reduce inflammation; relieve cough and reduce phlegm	N
Ertong Huichun Granules (EHGs)	Guizhou Jingcheng Pharmaceutical Co. Ltd.	1. Sojae Semen Praeparatum, 25g;<br>2. Schizonepetae Herba, 50g;<br>3. Puerariae Lobatae Radix, 50g;<br>4. Paeoniae Radix Rubra, 50g;<br>5. Scutellariae Radix, 50g;<br>6. Platycodonis Radix, 75g;<br>7. Bupleuri Radix, 37.5g;<br>8. Isatidis Folium, 50g;<br>9. Coptidis Rhizoma, 25g;<br>10. Powerdered Buffalo Horn Extract, 50g;<br>11. Saigae Tataricae Cornu, 25g;<br>12. Notopterygii Rhizoma et Radix, 50g;<br>13. Rehmanniae Radix, 50g;<br>14. Clematidis Armandii Caulis, 50g;<br>15. Peucedani Radix, 75g;<br>16. Scrophulariae Radix, 75g;<br>17. Tamaricis Cacumen, 37.5g;<br>18. Cimicifugae Rhizoma, 20g;<br>19. Arctii Fructus, 75g;<br>20. Praecipitatum Urinarium Hominis, 25g	1. *Glycine max* (L.) Merr;<br>2. *Schizonepeta tenuifolia* Eriq.;<br>3. *Pueraria lobata* (Willd.) Ohwi;<br>4. *Paeonia ladiflora* Pall. or *Paeonia veitchii* Lynch;<br>5. *Scutellaria baicalensis* Georgi;<br>6. *Platycodon grandiflorum* (Jacq.) A. DC;<br>7. *Bupleurum chinense* DC. or *Bupleurum scorzonerifolium* Willd.;<br>8. *Isatis indigotica* Fort.<br>9. *Coptis chinensis* Franch. or *Coptis deltoidea* C. Y. Cheng et Hsiao or *Coptis teeta* Wall.;<br>10. *Bubalus bubalis* Linnaens;<br>11. *Saiga tatarica* Linnaeu;<br>12. *Notopterygium incisum* Ting ex H. T. Chang or *Notopterygium franchetii* H. de Boiss.;<br>13. *Rehjnannia glutinosa* Libosch.;<br>14. *Clematis armandii* Franch. or *Clematis montana* Buch. -Ham.;<br>15. *Peucedanum praeruptorum* Dunn;<br>16. *Scrophularia ningpoensis* Hemsl.;<br>17. *Tamarix chinensis* Lour.;<br>18. *Cimicifuga heracleifolia* Kom. or *Cimicifuga dahurica* (Turcz.) Maxim. or *Cimicifuga foetida* L.;<br>19. *Arctium lappa* L.<br>20. *Homo sapiens* Linnaeus	**clear heat** and **remove toxin**; outthrust exterior syndrome and induce phlegm	Y - Prepared according to National Food and Drug Administration National Drug Standards. Standard number: WS_3-B-2276-97

Fangfeng Tongsheng Granules (FTGs)	Shandong Runzhong Pharmaceutical Co. Ltd.	1. Scutellariae Radix, 151g;<br>2. Platycodonis Radix, 151g;<br>3. Menthae Haplocalycis Herba, 75.5g;<br>4. Ephedrae Herba, 75.5g;<br>5. Rhei Radix et Rhizoma, 75.5g;<br>6. Gardeniae Fructus, 37.8g;<br>7. Gypsum Fibrosum, 151g;<br>8. Radix Alba, 75.5g;<br>9. Forsythiae Fructus, 75.5g;<br>10. Glycyrrhizae Radix et Rhizoma, 302g;<br>11. Saposhnikoviae Radix, 75.5g;<br>12. Schizonepetae Spica, 37.8g;<br>13. Natrii Sulfas, 75.5g;<br>14. Talcum, 453g;<br>15. Chuanxiong Rhizoma, 75.5g;<br>16. Angelicae Sinensis Radix, 75.5g;<br>*17.* Atractylodis Macrocephalae Rhizoma, 37.8g	1. *Scutellaria baicalensis* Georgi;<br>2. *Platycodon grandiflorum* (Jacq.) A. DC;<br>3. *Mentha haplocalyx* Briq.;<br>4. *Ephedra sinica* Stapf or *Ephedra intermedia* Schrenk et C. A. Mey or *Ephedra equisetina* Bge.;<br>5. *Rheum palmatum* L. or *Rheum tanguticum* Maxim. ex Balf. or *Rheum officinale* Baill.;<br>6. *Gardenia jasminoides* Ellis;<br>7. $CaSO_4 \cdot 2H_2O$;<br>8. *Paeonia lactiflora* Pall.;<br>9. *Forsythia suspensa* (Thunb.) Vahl;<br>10. *Glycyrrhiza uralensis* Fisch. or *Glycyrrhiza inflata* Bat. or *Glycyrrhiza glabra* L.;<br>11. *Saposhnikovia divaricata* (Turcz.) Schischk.;<br>12. *Schizonepeta tenuisfolia* Briq.;<br>13. $Na_2SO_4 \cdot 10H_2O$<br>14. $Mg_3(Si_4O_{10})(OH)_2$;<br>15. *Ligusticum chuanxiong* Hort.;<br>16. *Angelica sinensis* (Oliv.) Diels;<br>17. *Atractylodes macrocephala* Koidz.;	release exterior syndrome and relax the interior; **clear heat** and **remove toxin**	Y- Prepared according to Pharmacopoeia of PR China (2020 Volume I), p942-944
Houerhuan Xiaoyan Granules (HXGs)	Jiangxi Xinglin Baima Pharmaceutical Co., Ltd	1. Folium Archidendri Clypeariae, 1000g	1. *Archidendron clypearia* (Jack) I. C. Nielsen	**clear heat** and **remove toxin**; cool blood and detumescence; stop diarrhea	Y - Prepared according to National Food and Drug Administration National Drug Standards. Standard number: YBZ04122005-2009Z
Jinye Baidu Granules (JBGs) △	Sinopharm Zhonglian Pharmaceutical Co. Ltd	1. Lonicerae Japonicae Flos;<br>2. Isatidis Folium;<br>3. Taraxaci Herba;<br>4. Houttuyniae Herba	1. *Lonicera japonica* Thunb.;<br>2. *Isatis indigotica* Fort.;<br>3. *Taraxacum mongolicum* Hand.-Mazz. or *Taraxacum borealisinense* Kitam.;<br>4. *Houttuynia cordata* Thunb.	**clear heat** and **remove toxin**	N
Jinlianhua Granules (JLHGs)	Chengde Tianyuan Pharmaceutical Co., Ltd	1. Trollius Chinensis, 1000g	1. *Trollius chinensis* Bunge or *T. ledebouri* Reichb.	**clear heat** and **remove toxin**	Y- Prepared according to Pharmacopoeia of PR China (2020 Volume I), p1162-1163
Kanggan Granules (KangGs)	Sichuan GoodDoctor Panxi Pharmaceutical Co. Ltd.	1. Lonicerae Japonicae Flos, 700g;<br>2. Paeoniae Radix Rubra, 700g;<br>3. Dryopteridis Crassirhizomatis Rhizoma, 233g	1. *Lonicera japonica* Thunb.;<br>2. *Paeonia ladiflora* Pall. or *Paeonia veitchii* Lynch;<br>*3.* *Dryopteris crassirhizoma* Nakai	**clear heat** and **remove toxin**	Y - Prepared according to Pharmacopoeia of PR China (2020 Volume I), p1029-1030

Kouyanqing Granules (KouGs)	Guangzhou Baiyunshan Heji Huangpu Traditional Chinese Medicine Co. Ltd.	1. Asparagi Radix, 250g;<br>2. Ophiopogonis Radix, 250g;<br>3. Scrophulariae Radix, 250g;<br>4. Lonicerae Flos, 300g;<br>5. Glycyrrhizae Radix et Rhizoma 125g	1. *Asparagus cochinchinensis* (Lour.) Merr.;<br>2. *Ophiopogon japonicus* (L. f) Ker-Gawl.;<br>3. *Scrophularia ningpoensis* Hemsl.;<br>4. *Lonicera macranthoides* Hand. -Mazz. or *Lonicera hypoglauca* Miq. or *Lonicera confusa* DC. or *Lonicera fulvotomentosa* Hsu et S. C. Cheng;<br>*5.* *Glycyrrhiza uralensis* Fisch. or *Glycyrrhiza inflata* Bat. or *Glycyrrhiza glabra* L.	nourishing yin and **clear heat**; **remove toxin** and disperse swelling	Y - Prepared according to Pharmacopoeia of PR China (2020 Volume I), p529-530
Lianhua Qingwen Granules (LQGs)	Beijing Yiling Pharmaceutical Co. Ltd	1. Ephedrae Herba, 57g;<br>2. Rhei Radix et Rhizoma, 34g;<br>3. Gypsum Fibrosum, 170g;<br>4. Forsythiae Fructus, 170g;<br>5. Glycyrrhizae Radix et Rhizoma, 57g;<br>6. Lonicerae Japonicae Flos, 170g;<br>7. Armeniacae Semen Amarum, 57g;<br>8. Isatidis Radix, 170g;<br>9. Dryopteridis Crassirhizomatis Rhizoma, 170g;<br>10. Houttuyniae Herba, 170g;<br>11. Pogostemonis Herba, 57g;<br>12. Rhodiolae Crenulatae Radix et Rhizoma, 57g;<br>13. DL-Menthol, 5.0g	1. *Ephedra sinica* Stapf or *Ephedra intermedia* Schrenk et C. A. Mey or *Ephedra equisetina* Bge.;<br>2. *Rheum palmatum* L. or *Rheum tanguticum* Maxim. ex Balf. or *Rheum officinale* Baill.;<br>3. $CaSO_4 \cdot 2H_2O$;<br>4. *Forsythia suspensa* (Thunb.) Vahl;<br>5. *Glycyrrhiza uralensis* Fisch. or *Glycyrrhiza inflata* Bat. or *Glycyrrhiza glabra* L.;<br>6. *Lonicera japonica* Thunb.;<br>7. *Prunus armeniaca* L. var. ansu Maxim. or *Prunus sibirica* L. or *Prunus mandshurica* (Maxim.) Koehne or *Primus armeniaca* L.;<br>8. *Isat*is indigotica Fort.;<br>9. *Dryopteris crassirhizoma* Nakai;<br>10. *Houttuynia cordata* Thunb.;<br>11. *Pogostemon cablin* (Blanco) Benth.;<br>12. *Rhodiola crenulata* (Hook. f. et Thoms.) H. Ohba<br>13. $C_{10}H_{20}O$	clear seasonal febrile diseases and **remove toxin**; diffuse the lung and **purge heat**	Y- Prepared according to Pharmacopoeia of PR China (2020 Volume I), p1015-1016
Qingkailing Granules (QGs)	Guangzhou Baiyunshan Mingxing Pharmaceutical Co. Ltd. / Harbin Yizhou Pharmaceutical Co., Ltd	1. Margaritifera Concha, 200g;<br>2. Gardeniae Fructus, 100g;<br>3. Bubali Cornu, 100g;<br>4. Isatidis Radix, 800g;<br>5. Lonicerae Japonicae Flos, 240g;<br>6. Cholic acid, 13g;<br>7. Hyodeoxycholic acid, 15g;<br>8. Baicalin 20g	1. *Hyriopsis cumingii* (Lea) or *Cristaria plieata* (Leach) or *Pteria martensii* (Dunker);<br>2. *Gardenia jasminoides* Ellis;<br>3. *Bubalus bubalis* Linnaeu;<br>4. *Isatis indigotica* Fort.;<br>5. *Lonicera japonica* Thunb.;<br>6. $C_{24}H_{40}O_5$;<br>7. $C_{24}H_{40}O_4$;<br>*8.* $C_{21}H_{18}O_{11}$	**clear heat** and **remove toxi**n; tranquilizing and allaying excitement	Y - Prepared according to Pharmacopoeia of PR China (2020 Volume I), p1661-1662
Reduping Granules (RGs)	Jilin Jinbao Pharmaceutical Co., Ltd / Jiangxi Zhongshan Pharmaceutical Co. Ltd	1. Gypsum Fibrosum, 670g;<br>2. Lonicerae Japonicae Flos, 134g;<br>3. Scrophulariae Radix, 107g;<br>4. Rehmanniae Radix, 80g;<br>5. Forsythiae Fructus, 67g;<br>6. Gardeniae Fructus, 67g;<br>7. Gueldenstaedtiae Herba, 67g;<br>8. Scutellariae Radix, 67g;<br>9. Gentianae Radix et Rhizoma, 67g;<br>10. Isatidis Radix, 67g;<br>11. Anemarrhenae Rhizoma, 54g;<br>12. Ophiopogonis Radix 54g	1. $CaSO_4 \cdot 2H_2O$;<br>2. *Lonicera japonica* Thunb.;<br>3. *Scrophularia ningpoensis* Hemsl.;<br>4. *Rehjnannia glutinosa* Libosch.;<br>5. *Forsythia suspensa* (Thunb.) Vahl;<br>6. *Gardenia jasminoides* Ellis;<br>7. *Gueldenstaedtia verna*（Georgi）A. Bor.;<br>8. *Scutellaria baicalensis* Georgi;<br>9. *Gentiana manshurica* Kitag. or *Gentiana scabra* Bge. or *Gentiana triflora* Pall. or *Gentiana rigescens* Franch.;<br>10. *Isatis indigotica* Fort.;<br>11. *Anemarrhena asphodeloides* Bge.;<br>12. *Ophiopogon japonicus* (L. f) Ker-Gawl.	**clear heat** and **remove toxi**n	Y - Prepared according to National Food and Drug Administration National Drug Standards. Standard number: WS_3-B-3965-98-2

Shanlameiye Granules (SGs)	Jiangxi Youmei Pharmaceutical Co., Ltd.	1. *Chimonanthus nitens* Oliv., 1000g;<br>2. Saccharose, 600g;<br>3. Dextrin, 200g	1. *Calycanthus nitens* Rehd. or *Meratia nitens* (Oliv.) Rehd. et Wils.;<br>2. $C_{12}H_{22}O_{11}$;<br>3. $(C_6H_{10}O_5)n$	release exterior syndrome with pungent-cool; **clear heat** and **remove toxi**n	Y - Prepared according to National Food and Drug Administration National Drug Standards. Standard number: WS-11315(ZD-1315)-2002-2012Z
Xiao'er Chaigui Ture Granules (XCTGs)	Sunflower Pharmaceutical Group (Xiangyang) Longzhong Co., Ltd. / Guizhou Bailing Group Pharmaceutical Co., Ltd..	1. Puerariae Lobatae Radix, 260g;<br>2. Scutellariae Radix, 120g;<br>3. Bupleuri Radix, 260g;<br>4. Radix Alba, 90g;<br>5. Cinnamomi Ramulus, 90g;<br>6. Spirodelae Herba, 90g;<br>7. Cicadae Periostracum, 90g	1. *Pueraria lobata* (Willd.) Ohwi;<br>2. *Scutellaria baicalensis* Georgi;<br>3. *Bupleurum chinense* DC. or *Bupleurum scorzonerifolium* Willd.;<br>4. *Paeonia lactiflora* Pall.;<br>5. *Cinnamomum cassia* Presl;<br>6. *Spirodela polyrrhiza* (L.) Schleid;<br>7. *Cryptotympana pustulata* Fabricius	promote sweating to release exterior syndrome; clear the interior to **reduce heat**	Y - Prepared according to Pharmacopoeia of PR China (2020 Volume I), p575-576
Xiao'er Chiqiao Qingre Granules (XCQGs)	Jichuan Pharmaceutical Group Co. Ltd.	1. Sojae Semen Praeparatum, 333g;<br>2. Schizonepetae Herba, 222g;<br>3. Paeoniae Radix Rubra, 222g;<br>4. Scutellariae Radix, 333g;<br>5. Bupleuri Radix, 222g;<br>6. Menthae Haplocalycis Herba, 222g;<br>7. Rhei Radix et Rhizoma, 189g;<br>8. Gardeniae Fructus, 189g;<br>9. Forsythiae Fructus, 444g;<br>10. Glycyrrhizae Radix et Rhizoma, 189g;<br>11. Artemisiae Annuae Herba, 333g;<br>12. Arecae Semen, 167g;<br>13. Magnoliae Officinalis Cortex, 333g;<br>14. Pinelliae Rhizoma, 333g	1. *Glycine max* (L.) Merr;<br>2. *Schizonepeta tenuifolia* Eriq.;<br>3. *Paeonia ladiflora* Pall. or *Paeonia veitchii* Lynch;<br>4. *Scutellaria baicalensis* Georgi;<br>5. *Bupleurum chinense* DC. or *Bupleurum scorzonerifolium* Willd.;<br>6. *Mentha haplocalyx* Briq.;<br>7. *Rheum palmatum* L. or *Rheum tanguticum* Maxim. ex Balf. or *Rheum officinale* Baill.;<br>8. *Gardenia jasminoides* Ellis;<br>9. *Forsythia suspensa* (Thunb.) Vahl;<br>10. *Glycyrrhiza uralensis* Fisch. or *Glycyrrhiza inflata* Bat. or *Glycyrrhiza glabra* L.;<br>11. *Artemisia annua* L.;<br>12. *Areca catechu* L.;<br>13. *Magnolia officinalis* Rehd. et Wils. or *Magnolia officinalis* Rehd. et Wils. var. biloba Rehd. et Wils.<br>14. *Pineilia ternata* (Thunb.) Breit	disperse wind and release exterior syndrome; **clear heat** and disperse stagnation	Y - Prepared according to Pharmacopoeia of PR China (2020 Volume I), p582-583
Xiao'er Jinqiao Granules (XJGs) △	Sichuan Kaijing Pharmaceutical Co. Ltd.	1. Puerariae Lobatae Radix;<br>2. Bupleuri Radix;<br>3. Forsythiae Fructus;<br>4. Glycyrrhizae Radix et Rhizoma;<br>5. Lonicerae Japonicae Flos;<br>6. Isatidis Folium;<br>7. Sophorae Tonkinensis Radix et Rhizoma	1. *Pueraria lobata* (Willd.) Ohwi;<br>2. *Bupleurum chinense* DC. or *Bupleurum scorzonerifolium* Willd.;<br>3. *Forsythia suspensa* (Thunb.) Vahl;<br>4. *Glycyrrhiza uralensis* Fisch. or *Glycyrrhiza inflata* Bat. or *Glycyrrhiza glabra* L.;<br>5. *Lonicera japonica* Thunb.;<br>6. *Isatis indigotica* Fort.;<br>7. *Sophora tonkinensis* Gagnep.	disperse wind and **clear heat**; **remove toxin** and disinhibit throat; disperse swelling and relieve pain	Y - Prepared according to National Food and Drug Administration National Drug Standards. Standard number: WS_3-893(Z-178)-2004(Z)

Xiao'er Resuqing Granules (XRGs)	Harbin Shengtai Biological Pharmaceutical Co., Ltd	1. Bupleuri Radix, 1250g;<br>2. Scutellariae Radix, 625g;<br>3. Isatidis Radix, 1250g;<br>4. Puerariae Lobatae Radix, 625g;<br>5. Lonicerae Japonicae Flos, 687.5g;<br>6. Bubali Cornu, 312.5g;<br>7. Forsythiae Fructus, 750g;<br>8. Rhei Radix et Rhizoma, 312.5g	1. *Bupleurum chinense* DC. or *Bupleurum scorzonerifolium* Willd.;<br>2. *Scutellaria baicalensis* Georgi;<br>3. *Isatis indigotica* Fort.;<br>4. *Pueraria lobata* (Willd.) Ohwi;<br>5. *Lonicera japonica* Thunb.;<br>6. *Bubalus bubalis* Linnaeu;<br>7. *Forsythia suspensa* (Thunb.) Vahl;<br>8. *Rheum palmatum* L. or *Rheum tanguticum* Maxim. ex Balf. or *Rheum officinale* Baill.	**clear heat**; **remove toxin**; benefit pharynx	Y - Prepared according to Pharmacopoeia of PR China (2020 Volume I), p578-579
Yanning Granules (YNGs)	Huizhou Jiuhui Pharmaceutical Co., Ltd	1. Herba Monochasmatis Savatieri, 500g;<br>2. Herba Hedyotidis, 250g;<br>3. Commelinae Herba, 250g	1. *Monochasma savatieri* Franchet ex Maximowicz;<br>2. *Hedyotis diffusa* Willd.;<br>3. *Commelina communis* L.	**clear heat** and **remove toxi**n; reduce inflammation and stop dysentery	Y - Prepared according to National Food and Drug Administration National Drug Standards. Standard number: WS_3-B-0956-91

Note:△protected variety of Chinese medicine without dosage information in detail; TCM, Traditional Chinese Medicine; The function of heat-clearing and detoxifying were marked as bold font.

Appendix S6

Direct pairwise meta-analysis of all outcomes for mild HFMD.

(A) Fever Clearance Time

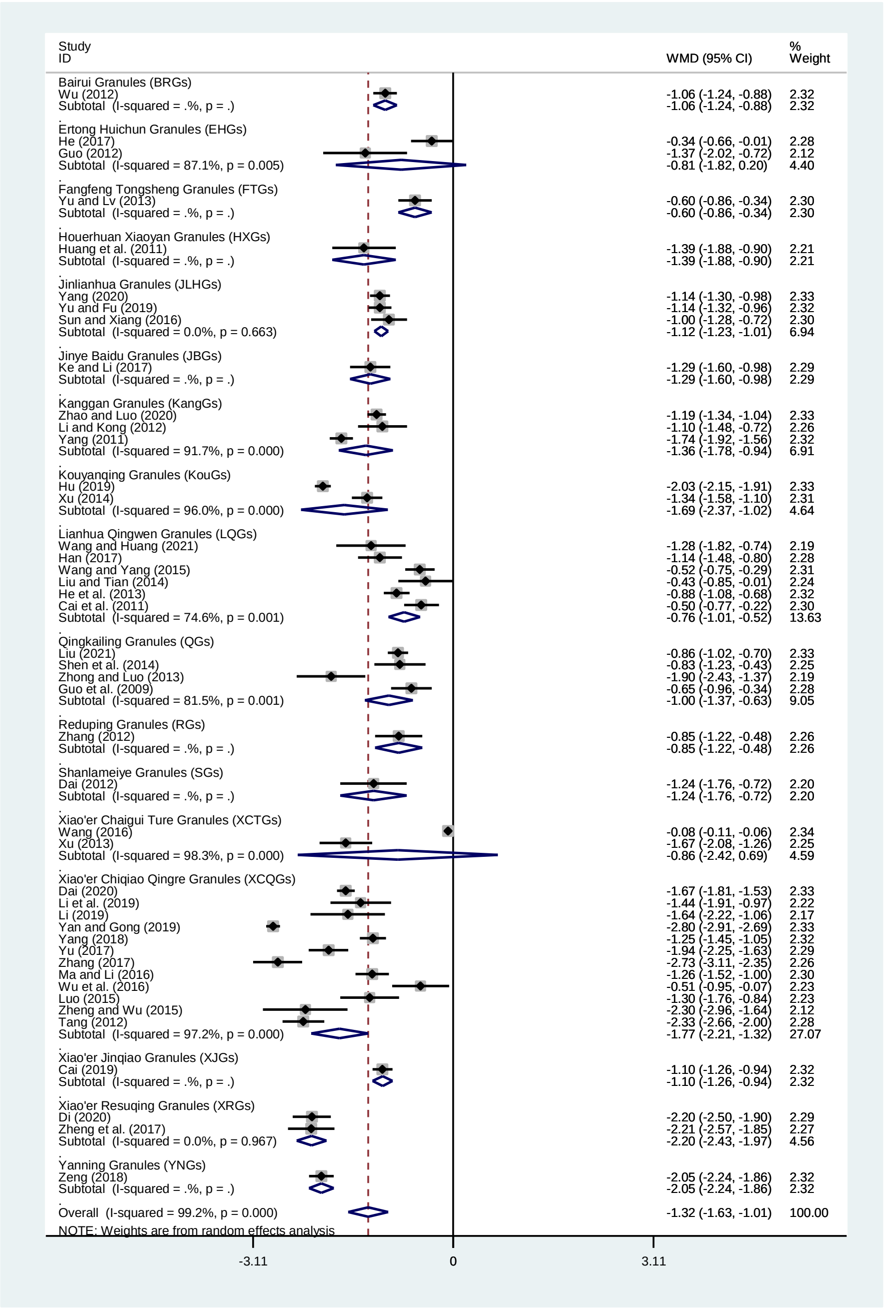

(B) Disappearance/scabbing time of rash

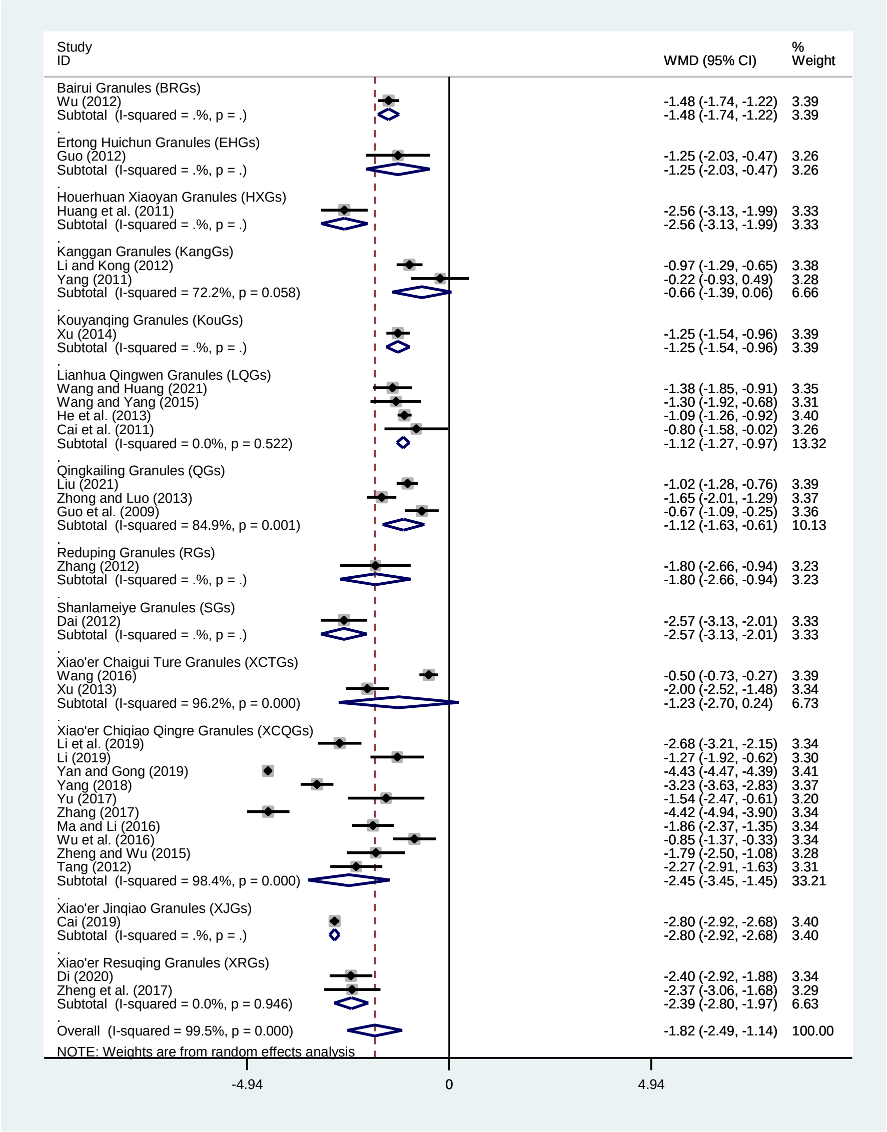

(C) Hospitalization/healing/treatment time

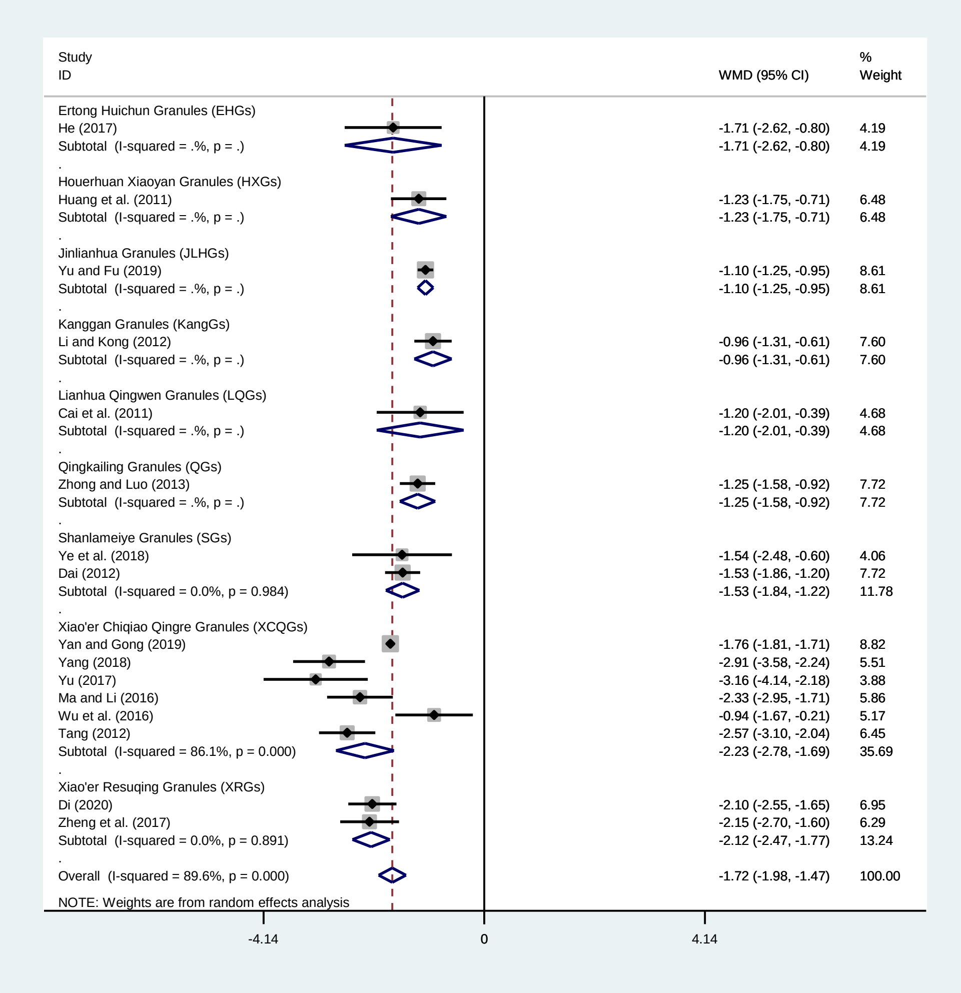

(D) Disappearance/Scabbing time of vesicles

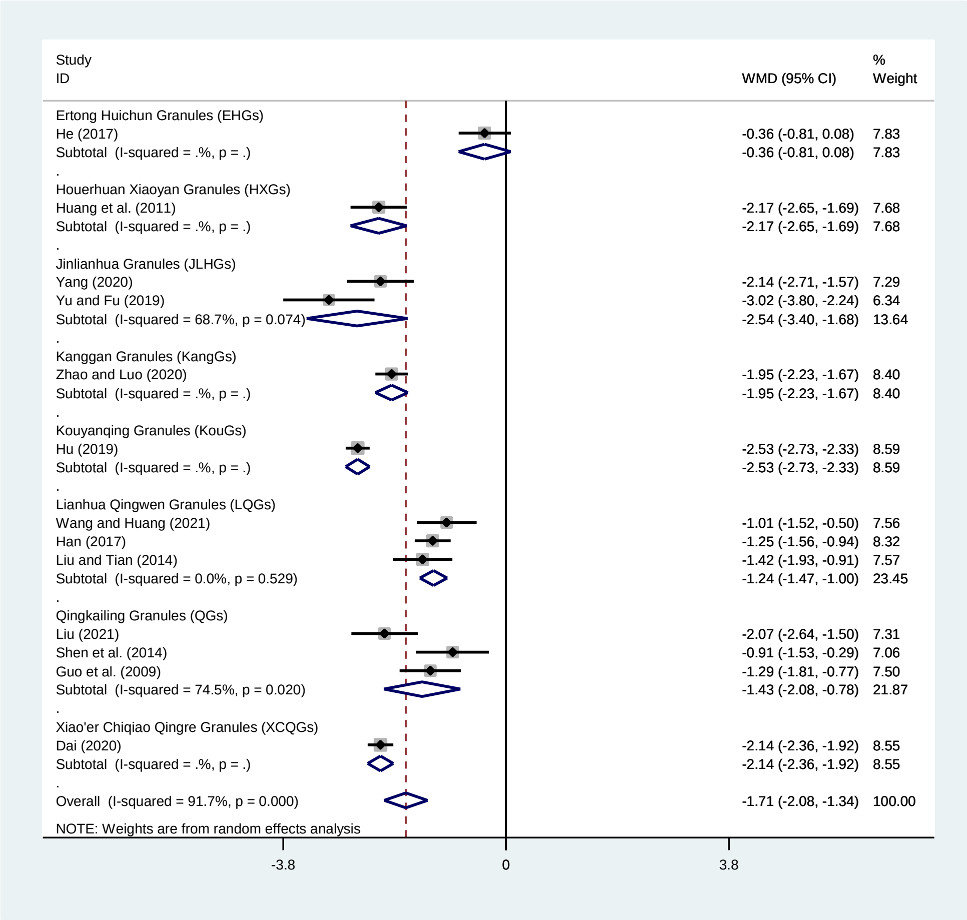

(E) Improvement time in appetite

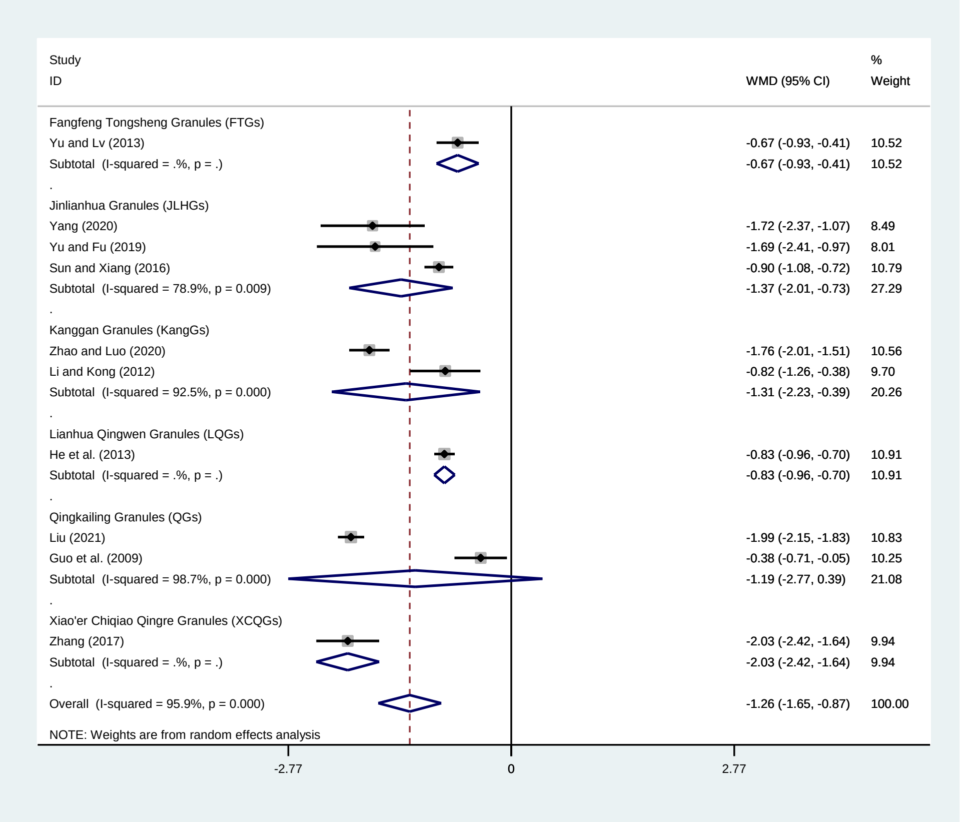

(F) Disappearance/healing time of ulcers

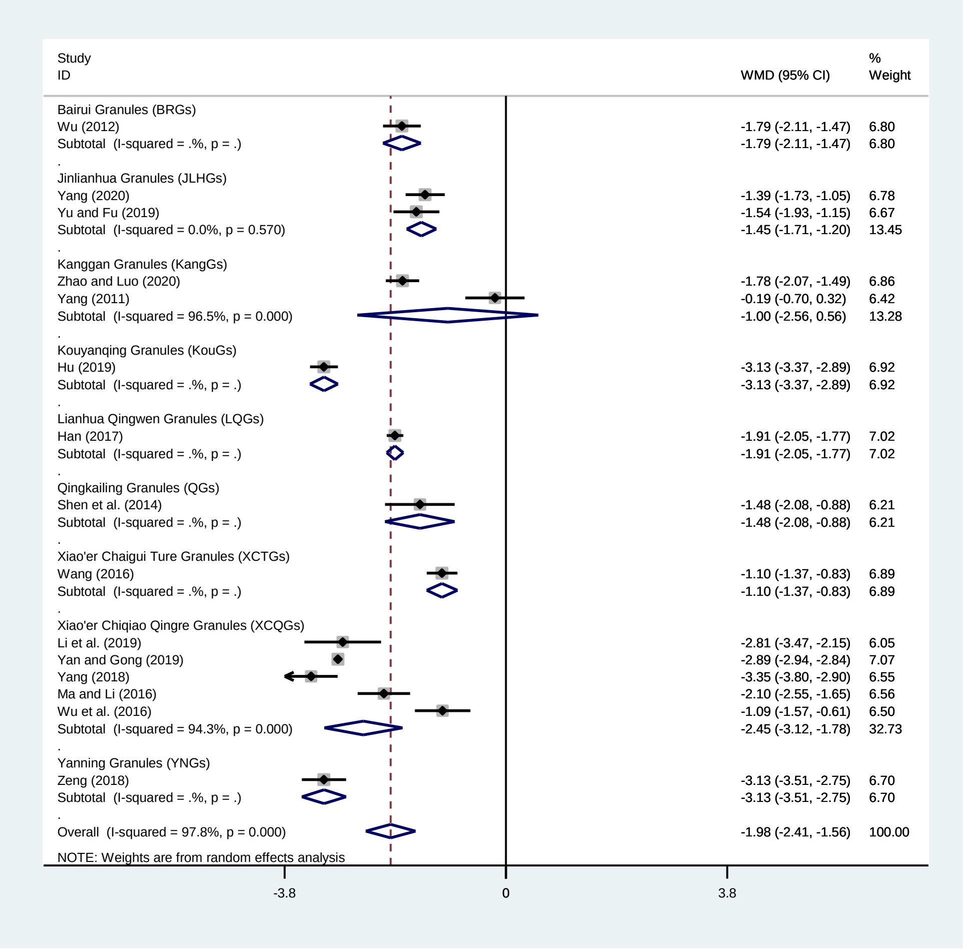

Figure S6-1 Results of primary outcomes with direct meta-analysis for mild HFMD.

(G) Total effectiveness rate (TER)

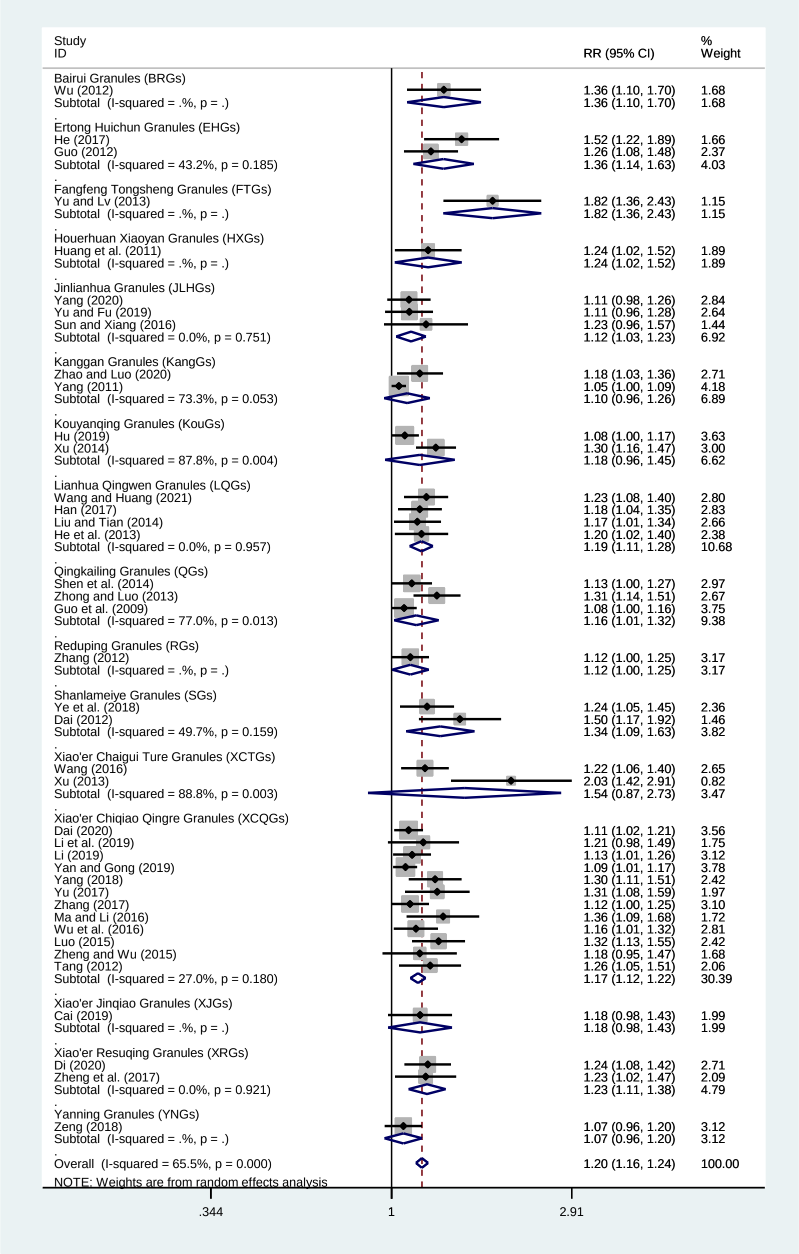

(H) Adverse effect rate (ADR)

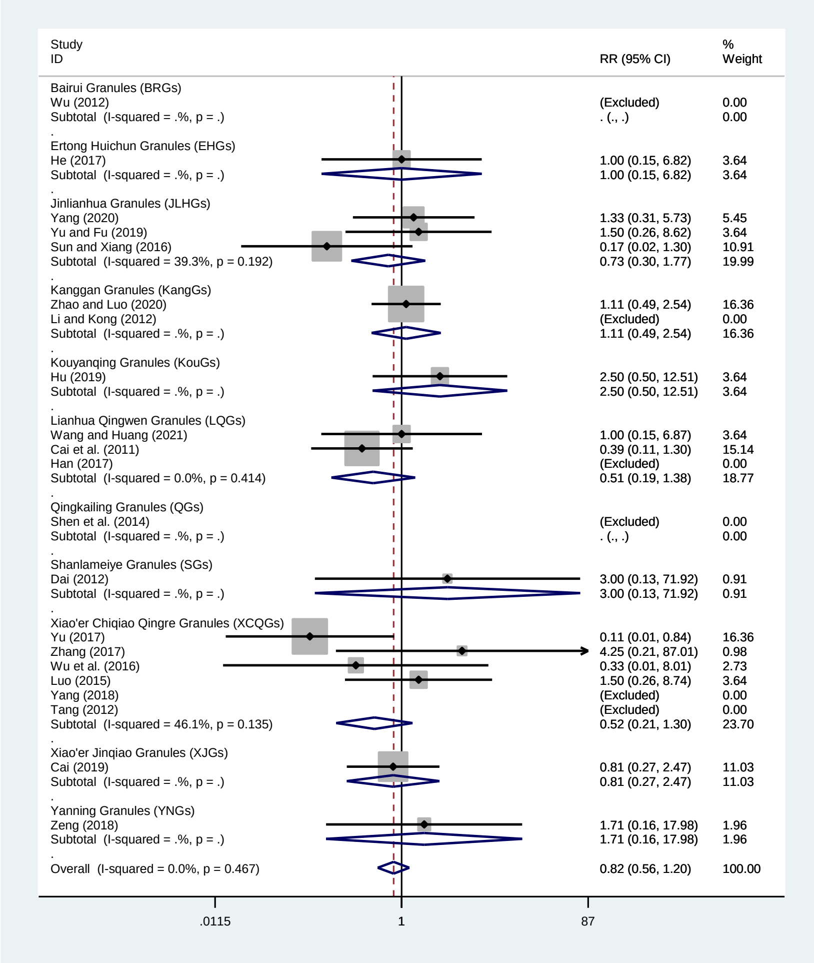

Figure S6-2 Results of secondary outcomes with direct meta-analysis for mild HFMD.

Appendix S7

Adverse reactions of HGs-HD in the included RCTs

Table S7. Adverse reactions of HGs-HD in the included RCTs

HGs-HD	Nausea [n (%)]		Vomiting [n (%)]		Diarrhea [n (%)]		Anorexia [n (%)]		Rash [n (%)]		Pruritus [n (%)]		Abnormal blood routine [n (%)]		Others [n (%)]		All [n (%)]		Total patients	
	T	C	T	C	T	C	T	C	T	C	T	C	T	C	T	C	T	C	T	C
**BRGs**	\	\	\	\	\	\	\	\	\	\	\	\	\	\	\	\	0 (0.0)	0 (0.0)	50	50
**EHGs**	\	\	\	\	2 (4.1)	2 (4.1)	\	\	\	\	\	\	\	\	\	\	2 (4.1)	2 (4.1)	49	49
**JLHGs**	\	\	\	\	\	\	\	\	3 (2.0)	2 (1.3)	3 (2.0)	2 (1.3)	\	\	2 (1.3)	7 (4.7)	8 (5.3)	11 (7.3)	150	150
**KangGs**	2 (1.8)	3 (2.8)	4 (3.6)	3 (2.8)	4 (3.6)	3 (2.8)	\	\	\	\	\	\	\	\	\	\	10 (9.0)	9 (8.4)	111	107
**KouGs**	3 (3.8)	1 (1.3)	2 (2.5)	0 (0.0)	\	\	\	\	\	\	0 (0.0)	1 (1.3)	\	\	\	\	5 (6.3)	2 (2.5)	80	80
**LQGs**	1 (0.7)	5 (3.2)	1 (0.7)	2 (1.3)	1 (0.7)	1 (0.6)	1 (0.7)	0 (0.0)	\	\	0 (0.0)	1 (0.6)	1 (0.7)	2 (1.3)	0 (0.0)	1 (0.6)	5 (3.3)	11 (7.1)	151	156
**QGs**	\	\	\	\	\	\	\	\	\	\	\	\	\	\	\	\	0 (0.0)	0 (0.0)	55	55
**SGs**	\	\	\	\	1 (2.0)	0 (0.0)	\	\	\	\	\	\	\	\	\	\	1 (2.0)	0 (0.0)	50	50
**XCQGs**	\	\	\	\	6 (1.7)	9 (2.6)	0 (0.0)	1 (0.3)	\	\	\	\	2 (0.6)	0 (0.0)	\	\	8 (2.2)	10 (2.9)	358	346
**XJGs**	\	\	\	\	\	\	\	\	\	\	\	\	\	\	5 (11.4)	6 (14.0)	5 (11.4)	6 (14.0)	44	43
**YNGs**	1 (2.9)	1 (3.3)	1 (2.9)	0 (0.0)	\	\	\	\	\	\	0 (0.0)	0 (0.0)	\	\	\	\	2 (5.7)	1 (3.3)	35	30
**Total**	7 (0.62)	10 (0.90)	8 (0.71)	5 (0.45)	14 (1.24)	15 (1.34)	1 (0.09)	1 (0.09)	3 (0.26)	2 (0.18)	3 (0.26)	4 (0.36)	3 (0.26)	2 (0.18)	7 (0.62)	14 (1.25)	46 (4.06)	52 (4.66)	1133	1116

Note: T, Test Group; C, Control Group; BRGs, Bairui Granules; EHGs, Ertong Huichun Granules; JLHGs, Jinlianhua Granules; KangGs, Kanggan Granules; KouGs, Kouyanqing Granules; LQGs, Lianhua Qingwen Granules; QGs, Qingkailing Granules; SGs, Shanlameiye Granules; XCQGs, Xiao'er Chiqiao Qingre Granules; XJGs, Xiao'er Jinqiao Granules; YNGs, Yanning Granules; “\” denotes “not included”.

Appendix S8

Radar map of ranking of treatment options relative to six primary outcomes based on SUCRA

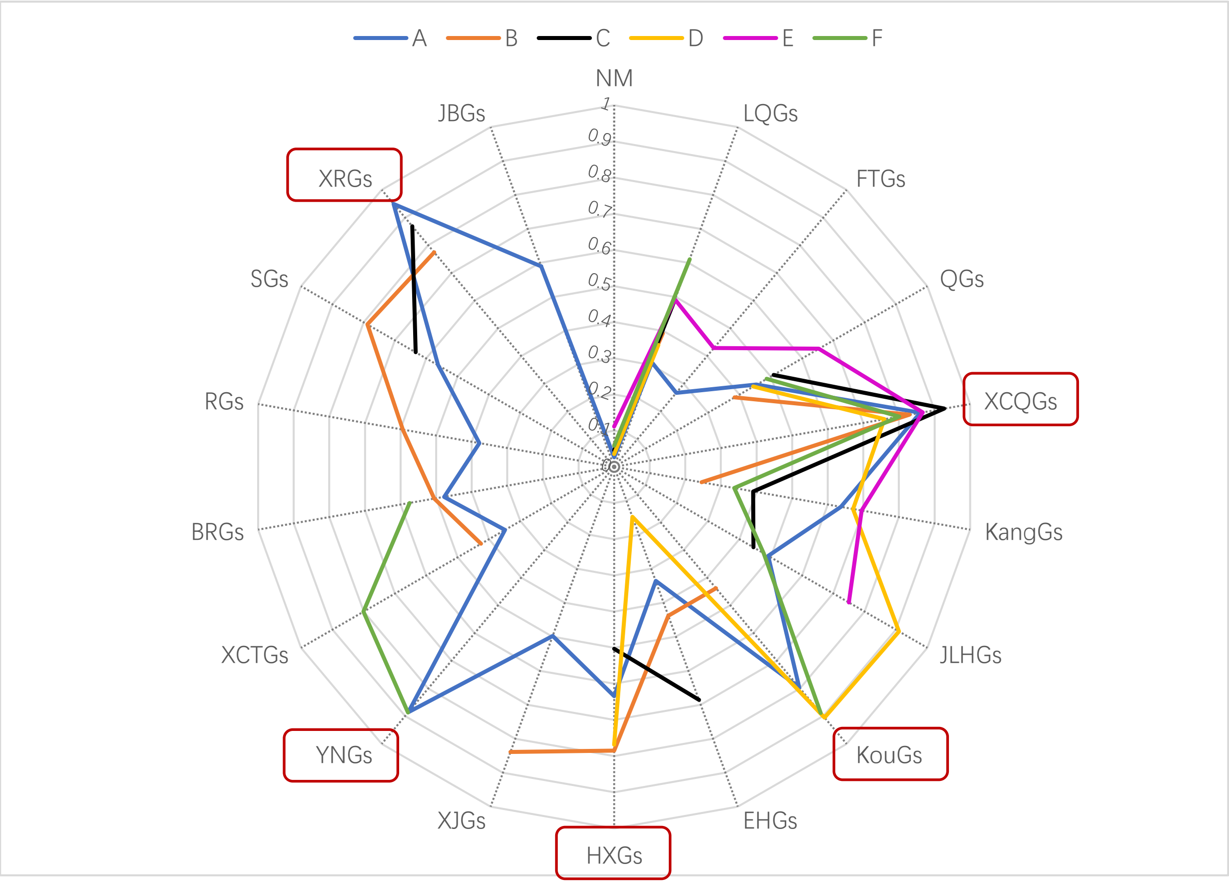

Figure S8 Radar map of ranking of treatment options relative to six primary outcomes based on SUCRA. (If the intervention resulted in a more favorable effect, then the point in certain outcome is close to the outside of the map; The top 5 HGs-HD in comprehensive rankings of symptom improvement effect by Inverse probability weighting adjustment were framed by red square.) A, Fever Clearance Time; B, Disappearance/scabbing time of rash; C, Hospitalization/healing/trceatment time; D, Disappearance/scabbing time of vesicles; E, Improvement time in appetite; F, Disappearance/healing time of ulcers; NM, No Medication; BRGs, Bairui Granules; EHGs, Ertong Huichun Granules; FTGs, Fangfeng Tongsheng Granules; HXGs, Houerhuan Xiaoyan Granules; JLHGs, Jinlianhua Granules; JBGs, Jinye Baidu Granules; KangGs, Kanggan Granules; KouGs, Kouyanqing Granules; LQGs, Lianhua Qingwen Granules; QGs, Qingkailing Granules; RGs, Reduping Granules; SGs, Shanlameiye Granules; XCTGs, Xiao'er Chaigui Ture Granules; XCQGs, Xiao'er Chiqiao Qingre Granules; XJGs, Xiao'er Jinqiao Granules; XRGs, Xiao'er Resuqing Granules; YNGs, Yanning Granules.

Appendix S9

Assessment of similarity results on mean age and mean course of disease among 45 studies

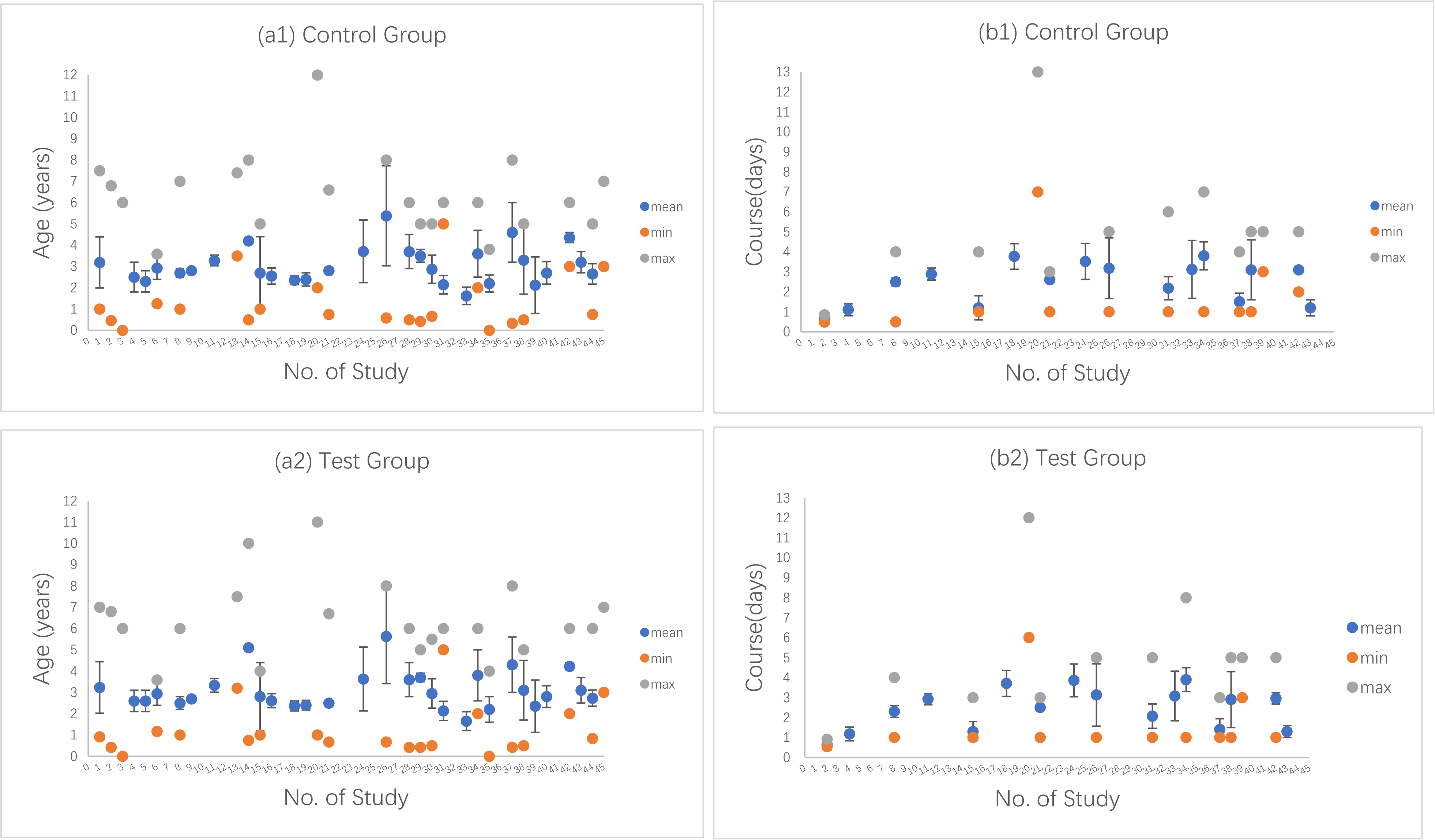

Figure S9. Assessment of similarity results on age and course on mild HFMD.

Appendix S10

Comparison-adjusted funnel plot for mild HFMD

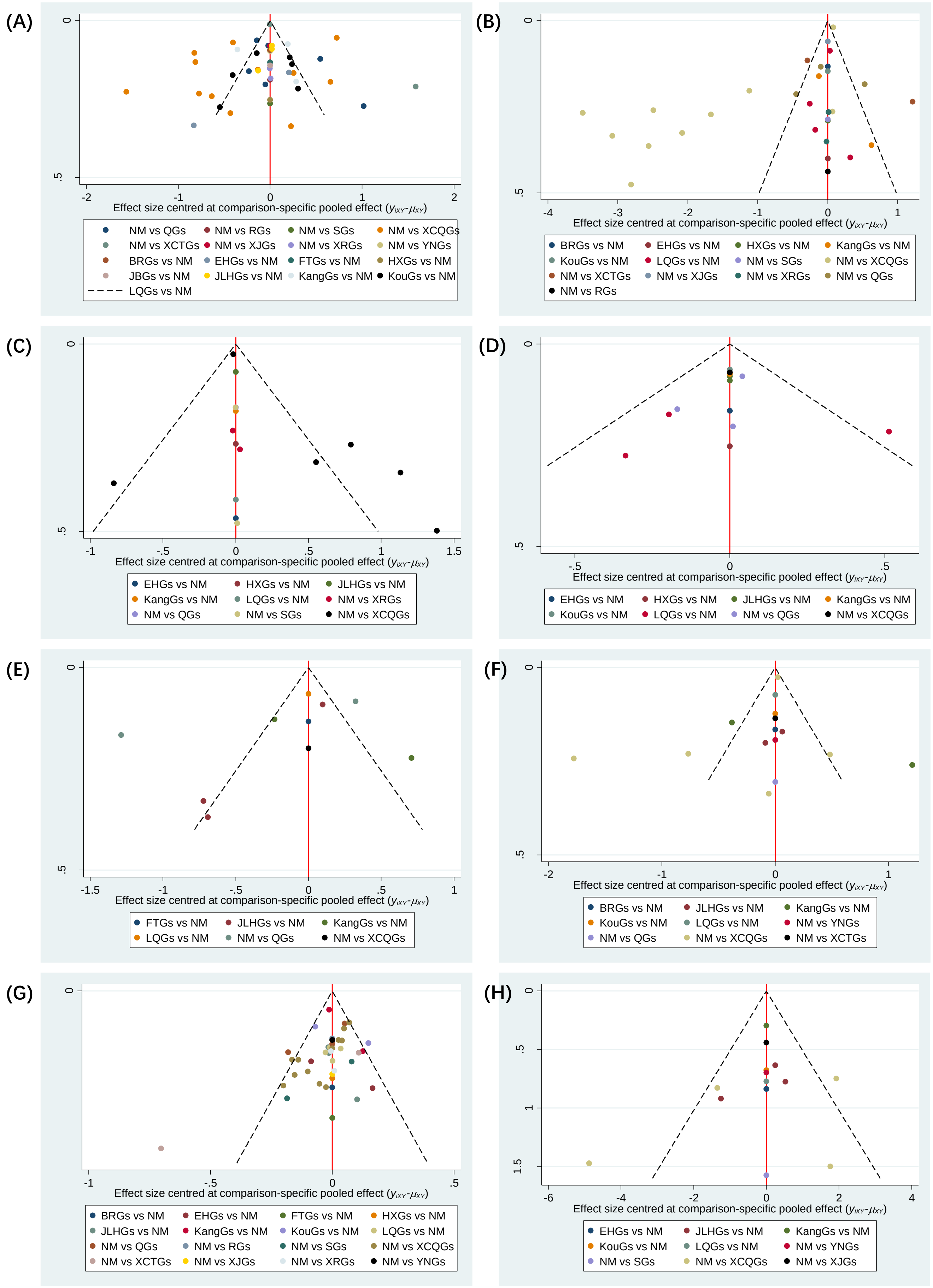

Figure S10 Comparison-adjusted funnel plot of HFMD. A, Fever Clearance Time; B, Disappearance/scabbing time of rash; C, Hospitalization/healing/treatment time; D, Disappearance/scabbing time of vesicles; E, Improvement time in appetite; F, Disappearance/healing time of ulcers; G, Total Effectiveness Rate; H, Adverse effect Rate; NM, No Medication; BRGs, Bairui Granules; EHGs, Ertong Huichun Granules; FTGs, Fangfeng Tongsheng Granules; HXGs, Houerhuan Xiaoyan Granules; JLHGs, Jinlianhua Granules; JBGs, Jinye Baidu Granules; KangGs,

Kanggan Granules; KouGs, Kouyanqing Granules; LQGs, Lianhua Qingwen Granules; QGs, Qingkailing Granules; RGs, Reduping Granules; SGs, Shanlameiye Granules; XCTGs, Xiao'er Chaigui Ture Granules; XCQGs, Xiao'er Chiqiao Qingre Granules; XJGs, Xiao'er Jinqiao Granules; XRGs, Xiao'er Resuqing Granules; YNGs, Yanning Granules.

Appendix S11

PRISMA NMA Checklist

Section/Topic	Item #	Checklist Item	Reported on Page #
**TITLE**			
Title	1	Identify the report as a systematic review *incorporating a network meta-analysis (or related form of meta-analysis).*	1
**ABSTRACT**			
Structured summary	2	Provide a structured summary including, as applicable:<br>**Background:** main objectives<br>**Methods:** data sources; study eligibility criteria, participants, and interventions; study appraisal; and *synthesis methods, such as network meta-analysis.*<br>**Results:** number of studies and participants identified; summary estimates with corresponding confidence/credible intervals; *treatment rankings may also be discussed. Authors may choose to summarize pairwise comparisons against a chosen treatment included in their analyses for brevity.*<br>**Discussion/Conclusions:** limitations; conclusions and implications of findings.<br>**Other:** primary source of funding; systematic review registration number with registry name.	1
**INTRODUCTION**			
Rationale	3	Describe the rationale for the review in the context of what is already known, *including mention of why a network meta-analysis has been conducted.*	2
Objectives	4	Provide an explicit statement of questions being addressed, with reference to participants, interventions, comparisons, outcomes, and study design (PICOS).	2
**METHODS†**			
Protocol and registration	5	Indicate whether a review protocol exists and if and where it can be accessed (e.g., Web address); and, if available, provide registration information, including registration number.	NAV
Eligibility criteria	6	Specify study characteristics (e.g., PICOS, length of follow-up) and report characteristics (e.g.,	2

		years considered, language, publication status) used as criteria for eligibility, giving rationale. *Clearly describe eligible treatments included in the treatment network, and note whether any have been clustered or merged into the same node (with justification).*	
Information sources	7	Describe all information sources (e.g., databases with dates of coverage, contact with study authors to identify additional studies) in the search and date last searched.	2-3
Search	8	Present full electronic search strategy for at least one database, including any limits used, such that it could be repeated.	2-3, Appendix S1
Study selection	9	State the process for selecting studies (i.e., screening, eligibility, included in systematic review, and, if applicable, included in the meta-analysis).	3
Data collection process	10	Describe method of data extraction from reports (e.g., piloted forms, independently, in duplicate) and any processes for obtaining and confirming data from investigators.	3
Data items	11	List and define all variables for which data were sought (e.g., PICOS, funding sources) and any assumptions and simplifications made.	3
**Geometry of the network**	**S1**	Describe methods used to explore the geometry of the treatment network under study and potential biases related to it. This should include how the evidence base has been graphically summarized for presentation, and what characteristics were compiled and used to describe the evidence base to readers.	3, Figure 3
Risk of bias within individual studies	12	Describe methods used for assessing risk of bias of individual studies (including specification of whether this was done at the study or outcome level), and how this information is to be used in any data synthesis.	3
Summary measures	13	State the principal summary measures (e.g., risk ratio, difference in means). *Also describe the use of additional summary measures assessed, such as treatment rankings and surface under the cumulative ranking curve (SUCRA) values, as well as modified approaches used to present summary findings from meta-analyses.*	3
Planned methods of analysis	14	Describe the methods of handling data and combining results of studies for each network meta-analysis. This should include, but not be limited to:<br>• *Handling of multi-arm trials;*<br>• *Selection of variance structure;*<br>• *Selection of prior distributions in Bayesian analyses; and*<br>• *Assessment of model fit.*	3
**Assessment of Inconsistency**	**S2**	Describe the statistical methods used to evaluate the agreement of direct and indirect evidence in	3

		the treatment network(s) studied. Describe efforts taken to address its presence when found.	
Risk of bias across studies	15	Specify any assessment of risk of bias that may affect the cumulative evidence (e.g., publication bias, selective reporting within studies).	4
Additional analyses	16	Describe methods of additional analyses if done, indicating which were pre-specified. This may include, but not be limited to, the following:<br>• Sensitivity or subgroup analyses;<br>• Meta-regression analyses;<br>• *Alternative formulations of the treatment network; and*<br>• *Use of alternative prior distributions for Bayesian analyses (if applicable).*	3
**RESULTS†**			
Study selection	17	Give numbers of studies screened, assessed for eligibility, and included in the review, with reasons for exclusions at each stage, ideally with a flow diagram.	4, Figure 1
**Presentation of network structure**	**S3**	Provide a network graph of the included studies to enable visualization of the geometry of the treatment network.	4, Figure 3
**Summary of network geometry**	**S4**	Provide a brief overview of characteristics of the treatment network. This may include commentary on the abundance of trials and randomized patients for the different interventions and pairwise comparisons in the network, gaps of evidence in the treatment network, and potential biases reflected by the network structure.	4, Appendix S9
Study characteristics	18	For each study, present characteristics for which data were extracted (e.g., study size, PICOS, follow-up period) and provide the citations.	4, Appendix S2, Appendix S3, Appendix S4, Appendix S5
Risk of bias within studies	19	Present data on risk of bias of each study and, if available, any outcome level assessment.	5, Figure 2
Results of individual studies	20	For all outcomes considered (benefits or harms), present, for each study: 1) simple summary data for each intervention group, and 2) effect estimates and confidence intervals. *Modified approaches may be needed to deal with information from larger networks.*	4-5, Appendix S3
Synthesis of results	21	Present results of each meta-analysis done, including confidence/credible intervals. *In larger networks, authors may focus on comparisons versus a particular comparator (e.g. placebo or standard care), with full findings presented in an appendix. League tables and forest plots may be considered to summarize pairwise comparisons.*	5-10, Table 1, Table 2, Figure 4, Appendix S6, Appendix S7,

		If additional summary measures were explored (such as treatment rankings), these should also be presented.	Appendix S8
**Exploration for inconsistency**	**S5**	Describe results from investigations of inconsistency. This may include such information as measures of model fit to compare consistency and inconsistency models, *P* values from statistical tests, or summary of inconsistency estimates from different parts of the treatment network.	11
Risk of bias across studies	22	Present results of any assessment of risk of bias across studies for the evidence base being studied.	11, Appendix S10
Results of additional analyses	23	Give results of additional analyses, if done (e.g., sensitivity or subgroup analyses, meta-regression analyses, *alternative network geometries studied, alternative choice of prior distributions for Bayesian analyses*, and so forth).	5-10
**DISCUSSION**			
Summary of evidence	24	Summarize the main findings, including the strength of evidence for each main outcome; consider their relevance to key groups (e.g., healthcare providers, users, and policy-makers).	11
Limitations	25	Discuss limitations at study and outcome level (e.g., risk of bias), and at review level (e.g., incomplete retrieval of identified research, reporting bias). *Comment on the validity of the assumptions, such as transitivity and consistency. Comment on any concerns regarding network geometry (e.g., avoidance of certain comparisons).*	12
Conclusions	26	Provide a general interpretation of the results in the context of other evidence, and implications for future research.	13
**FUNDING**			
Funding	27	Describe sources of funding for the systematic review and other support (e.g., supply of data); role of funders for the systematic review. This should also include information regarding whether funding has been received from manufacturers of treatments in the network and/or whether some of the authors are content experts with professional conflicts of interest that could affect use of treatments in the network.	13

Note:

①PICOS = P–patient, problem or population; I–intervention; C–comparison, control or comparator; O–outcome; S–study design.

②Text in italics indicates wording specific to reporting of network meta-analyses that has been added to guidance from the PRISMA statement.

③"†"- Authors may wish to plan for use of appendices to present all relevant information in full

detail for items in this section.
④“NA”- Not applicable.
⑤“NAV”- Not available.
